# Co-infection and interaction of enteric pathogens in acute diarrhea among children under five years: a large-scale multicenter observational study from China

**DOI:** 10.1186/s40249-025-01392-8

**Published:** 2025-12-02

**Authors:** Shun-Xian Zhang, Qin-Yan Zuo, Jin-Xin Zheng, Ji-Chun Wang, Mu-Xin Chen, Yu Qin, Jian Yang, Shan Lv, Lei Duan, Li-Guang Tian, Qin Liu, Wen-Wen Lv, Rui-Tao Liu, Guang-Hua Chen, Wan-Fu Xu, Can-Jun Zheng, Shi-Zhu Li, Hong-Li Wang

**Affiliations:** 1 Longhua Hospital, Shanghai University of Traditional Chinese Medicine, Shanghai, 200032 China; 2 National Institute of Parasitic Diseases, Chinese Center for Disease Control and Prevention (Chinese Center for Tropical Diseases Research); National Key Laboratory of Intelligent Tracking and Forecasting for Infectious Diseases; NHC Key Laboratory of Parasite and Vector Biology; WHO Collaborating Centre for Tropical Diseases; National Center for International Research On Tropical Diseases, Ministry of Science and Technology, Shanghai, 200025 China; 3 National Key Laboratory of Intelligent Tracking and Forecasting for Infectious Diseases, Chinese Center for Disease Control and Prevention, Beijing, 102206 China; 4 Clinical Research Institute, Shanghai Jiao Tong University School of Medicine, Shanghai, 200025 China; 5 Guangzhou Women and Children’s Medical Center, Guangzhou Medical University, Guangzhou, 510623 People’s Republic of China

**Keywords:** Diarrheal disease, Children, Co-infection, Enteric pathogens, Synergistic interactions

## Abstract

**Background:**

Diarrhea remains a major health concern in children under five years, with enteric pathogens being key contributors. However, the interactions among these pathogens and their combined effects on disease severity are not well understood. The study investigates the interactions among co-infecting enteric pathogens on diarrhea pathogenesis within an epidemiological framework.

**Methods:**

This large-scale, multicenter case-control study was conducted from January 1, 2024 to December 31, 2024, across four tertiary hospitals in Guangzhou, Guangdong Province, China. Stool samples were collected from children under five years with diarrhea (cases) and those without (non-diarrheal children). 21 enteric pathogens in each specimen were identified. Potential interactions between co-infecting pathogens were assessed using both additive and multiplicative models.

**Results:**

Enteric pathogens were more frequently detected in children with diarrhea than in non-diarrheal children (53.6% vs. 27.8%, *P* < 0.001), with significantly higher detection of both viral (23.3% vs. 13.3%) and bacterial pathogens (34.2% vs. 12.5%). Pathogens independently associated with diarrhea included diarrheagenic *Escherichia coli* (DEC), *Vibrio parahaemolyticus*, *Clostridioides difficile* (CD), group A rotavirus (RVA), and Norovirus GII (NoVs GII). The proportion of children with any form of co-infection was also significantly higher in the diarrhea group compared with non-diarrheal children (16.1% vs. 4.5%, *χ*^2^ = 32.594, *P* < 0.001). Several specific dual-pathogen combinations—namely RVA + DEC (*χ*^2^ = 4.956, *P* = 0.026), RVA + CD (*χ*^2^ = 10.313, *P* < 0.001), RVA + NoVs GII (*χ*^2^ = 15.503, *P* < 0.001), and DEC + *Blastocystis hominis* (Bh)—were significantly more common among diarrhea cases (*χ*^2^ = 4.207, *P* = 0.041). Multiplicative interaction analysis further identified significant synergistic effects for RVA + DEC [odds ratio (*OR*) = 2.304, 95% confidence interval (*CI*): 1.194–5.089], RVA + CD (*OR* = 6.199, 95% *CI:* 1.701–10.601), RVA + NoVs GII (*OR* = 6.296, 95% *CI:* 2.061–10.723), and DEC + Bh (*OR* = 4.602, 95% *CI:* 2.213–9.878).

**Conclusion:**

This study demonstrates the frequent occurrence of co-infections in diarrheal children, and enteric pathogens may interact synergistically or antagonistically. It highlights the central role of RVA in exacerbating the severity of these co-infections. The findings emphasize the critical role of RVA vaccination in alleviating the burden and severity of diarrhea.

*Trial Registration* The study was registered in the Chinese Clinical Trial Registry (ChiCTR-ROC-17013620).

**Graphical Abstract:**

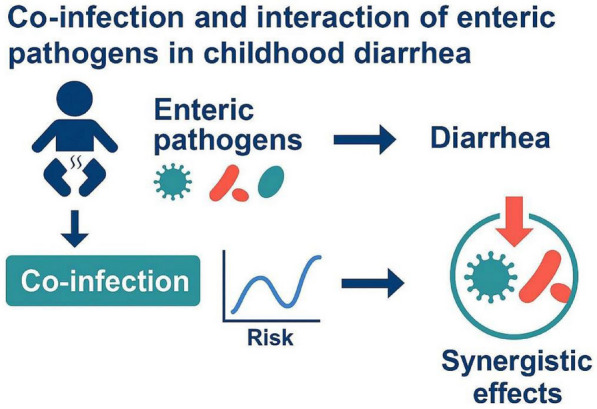

**Supplementary Information:**

The online version contains supplementary material available at 10.1186/s40249-025-01392-8.

## Background

Diarrhea disease is defined as the passage of three or more loose or liquid stools within a 24-h period and represents a common manifestation of gastrointestinal dysfunction [1, 2]. It can be caused by various factors, including exposure to unsafe water and food, poor hygiene practices, and frequent contact with animals harboring enteric pathogens. Among these, infection with enteric pathogens is the primary cause of diarrhea, Common causative agents include diarrheagenic *Escherichia coli* (DEC), non-typhoidal *Salmonella* spp.(NTS), *Shigella* spp., *Vibrio cholerae*, group A rotavirus (RVA), Norovirus (NoVs), *Entamoeba histolytica*, *Cryptosporidium*, *Giardia lamblia*, etc [1, 3].

Although the overall burden of diarrhea has significantly decreased over the past three decades— with incidence dropping from 190,036 per 100,000 population in 1990 to 59,677 per 100,000 population in 2021, and mortality falling from 263.95 to 51.72 per 100,000 population— it remains a significant global public health challenge, particularly among children [1, 2]. According to Global Burden of Disease (GBD) 2021 Study, the annual incidence of diarrhea among children under five years of age was 59,677.27 per 100,000 population [95% uncertainty interval (UI): 49,246.22–70,442.60], with a mortality rate of 51.72 per 100,000 population (95% UI: 38.13–70.54). Diarrhea has become the second leading cause of death among children under five, accounting for 26.93% of all deaths. Furthermore, nearly 90% of these deaths occur in South Asia and sub-Saharan Africa [1, 2].

Complex interactions persist between the gut microbiota and the human host [4]. Extensive host-pathogen interactions often arise due to high genetic variability in host resistance and immune function, alongside the diversity in pathogen infectivity and virulence [5]. Some enteric microbes coexist with the host in a state of mutual tolerance and co-evolutionary balance, wherein the host develops resistance and tolerance mechanisms, while the microbes evolve potent pathogenic strategies. Most gut microbes entering the host via the fecal-oral route do not cause clinical symptoms; however, a subset can lead to overt infections. Pathogenic microorganisms may adhere to the surface of intestinal epithelial cells, and their presence or metabolites can penetrate these cells, disrupting normal physiological functions and inducing pathological damage. Importantly, the host immune response to these microbes is also a major contributor to diarrheal symptoms [6]. Thus, the occurrence, clinical manifestations, and severity of diarrhea are determined by the interplay between gut microbes and the host, as well as the timing of medical intervention [7]. These complex dynamics result in considerable heterogeneity in clinical outcomes among patients infected with different pathogens—ranging from fever, nausea, vomiting, dehydration, and metabolic acidosis to, in severe cases, shock and death.

In nature, co-infection with multiple pathogens is a common phenomenon. Pathogens involved in co-infections may interact with each other as well as with the host, resulting in complex biological interplay [7, 8]. These interactions can be either synergistic (enhancing pathogenicity) or antagonistic (inhibiting effects). Interactions between coexisting pathogens can lead to immune suppression, whereby the primary pathogen weakens host defenses and facilitates secondary infections, resulting in synergistic effects. Such interactions may exacerbate disease severity and increase the risk of adverse outcomes, including mortality. For instance, co-infection with RVA and NoVs has been shown to increase the frequency of diarrheal episodes [9], while RVA co-infection with *Giardia lamblia* has been associated with greater disease severity [7]. In contrast, antagonistic interactions may arise from direct competition for resources or sites of infection, it can shape pathogen populations or alter infection dynamics. In some cases, the immune response elicited by the first pathogen may inhibit the colonization or replication of a second pathogen [10]. For example, RVA has been reported to exhibit a negative association with most other enteric pathogens, potentially due to competition for resources, immune-mediated inhibition, or interference by viral proteins that suppress the growth of co-infecting organisms [11, 12].

A deeper understanding of the complex interactions among enteric viruses, bacteria, and parasites is essential to elucidate the epidemiology of diarrheal pathogens and to inform the development of effective public health strategies for infection control. Currently, research on the interactions between bacterial, viral, and parasitic pathogens in acute diarrhea among children under five remains limited, and data on the causal association between these pathogens and acute diarrhea in this vulnerable population is scarce. A large-scale, hospital-based, multicenter case-control study involving children with and without acute diarrhea was conducted. The study aimed to elucidate the epidemiological patterns and causal relationships between diarrhea and specific enteric pathogens, and to assess whether these pathogens co-occur by chance or are structured within microbial communities. In addition, we sought to better understand the relationship between co-infections and diarrheal disease severity.

## Methods

### Study design and setting

This hospital-based, large-scale, multicenter case–control study was conducted in Guangzhou City, Guangdong Province, located in Southern China, between January 1, 2024 and December 31, 2024. Guangzhou lies within the geographic coordinates of 112°57′–114°3′E and 22°26′–23°56′N, situated at the Pearl River Delta estuary and bordering the South China Sea. The region experiences a subtropical monsoon climate, with an average elevation of 6.6 m, annual mean temperatures ranging from 21.5 ℃ to 22.2 ℃, and an average annual precipitation exceeding 1800 mm across approximately 150 rainy days. As of the end of 2023, the city had a permanent resident population of 18.73 million, with over 86% residing in urban areas [13].

The sentinel hospitals included in this study were not randomly selected from all hospitals in Guangzhou City. Instead, selection was based on multiple practical considerations, including high pediatric patient volumes, existing clinical and laboratory collaboration, and strong diagnostic capacity. Ultimately, four hospitals were included: Guangzhou Women and Children’s Medical Center, Guangzhou Children’s Hospital, Guangzhou Maternity and Child Health Care Hospital, and Zengcheng District Maternity and Child Health Hospital. Informed consent was obtained from each children’s parents or legal guardians. Furthermore, the study adhered to the guidelines outlined in the previous study [14].

### Definitions

Diarrhea was defined as the passage of three or more abnormal stools (e.g., watery, bloody, or mucoid) within a 24-h period. Acute diarrhea was characterized by the onset of ≥ 3 loose or watery stools per day with a disease duration of fewer than 14 days, measured from symptom onset to the time of clinical presentation [9]. Co-infection (or multiple infections) was defined as the concurrent detection of two or more diarrheagenic microorganisms in a single host [15]. Single infection (or mono-infection) referred to the presence of only one enteric pathogen included in this study within a single host [8].

Synergistic effect refers to a phenomenon in which the combined impact of two or more factors (e.g., pathogens) exceeds the sum of their individual effects. In the context of enteric infections, synergy may manifest when co-infecting pathogens aggravate disease severity by enhancing the expression of virulence factors, disrupting the intestinal mucosal barrier, or triggering a heightened immune-inflammatory response. These interactions can intensify clinical symptoms, prolong the disease course, and increase both pathogenicity and transmissibility [16].

Antagonistic effect refers to a phenomenon in which the combined impact of multiple factors is less than the sum of their individual effects, and may even result in partial or complete suppression of one factor’s activity. In the context of microbial infections, antagonism may arise due to competition for resources, immune-mediated suppression, or disruption of the microbial ecosystem. These interactions can attenuate the pathogenic potential of certain organisms and alter the clinical presentation of disease [7, 16].

For patients with acute diarrhea, the number of stools per day was used to assess the severity of the diarrheal episode. Specifically, severe diarrhea was defined as the passage of ≥ 6 liquid or semi-liquid stools within a 24-h period. Moderate diarrhea was defined as 4–5 such stools in the past 24 h, while mild diarrhea was defined as exactly 3 loose stools within the same timeframe [17, 18].

### Sampling and study participants

From January 1, 2024 to December 31, 2024, children with and without acute diarrhea were recruited from the outpatient or inpatient departments of designated voluntary sentinel hospitals. After trained physicians or nurses introduced the study objectives to the parents or legal guardians of eligible children, participants were selected using a two-stage sampling approach. First, one weekday (Monday) was randomly selected from Monday to Friday as the designated recruitment day. Cluster sampling was implemented at each of the four sentinel hospitals. All children under five years of age presenting with diarrhea to the gastrointestinal clinics or admitted to gastrointestinal wards were enrolled as potential cases. Concurrently, children under five visiting or admitted to general pediatrics, respiratory medicine, or hematology departments were screened as potential non-diarrheal subjects. Participants from both groups were further assessed for eligibility based on predefined inclusion and exclusion criteria.

Children with diarrhea were eligible for inclusion if they met the following criteria: (1) aged under five years, regardless of sex; (2) presented with acute diarrheal symptoms at the time of consultation; (3) had not used antibiotics within the preceding two weeks; (4) had not used probiotics within the preceding two weeks; (5) were recruited from gastrointestinal outpatient clinics or inpatient departments; and (6) had informed consent signed by a parent or legal guardian. Exclusion criteria included: (1) pre-existing gastrointestinal diseases; (2) malignancies or hematologic disorders; (3) current immunosuppressive therapy; (4) corticosteroid treatment; (5) incomplete or unrecoverable questionnaire data; (6) insufficient stool sample volume; (7) missing key clinical information (e.g., symptom presentation, number and duration of diarrheal episodes); and (8) concurrent participation in other clinical studies.

Children without diarrhea were eligible for inclusion as controls if they met the following criteria: (1) aged under five years, regardless of sex; (2) had not experienced any diarrheal illness in the two weeks prior to enrollment; (3) had not used antibiotics within the preceding two weeks; (4) had not used probiotics within the preceding two weeks; (5) were recruited from non-gastrointestinal outpatient or inpatient departments; and (6) had informed consent signed by a parent or legal guardian. Exclusion criteria included: (1) known gastrointestinal diseases; (2) malignancies or hematologic disorders; (3) current immunosuppressive therapy; (4) corticosteroid treatment; (5) insufficient stool sample volume; (6) missing key clinical information (e.g., symptom presentation, number or duration of stools); and (7) participation in other clinical studies.

### Sample size

This study employed a case–control design, with co-infection as the primary outcome of interest. Based on previous literature, the prevalence of enteric pathogen co-infections among children under five years of age with acute diarrhea is estimated at 15.0%, compared to 5.3% among non-diarrheal controls in the same age group [8]. Assuming a significance level (*α*) of 0.05 and power (1–*β*) of 0.90, the required sample size was calculated using the “Proportions – Tests for Two Proportions” module in Power Analysis and Sample Size Software (version 21, NCSS LLC, East Kaysville, Utah, USA). The analysis yielded a minimum of 199 participants per group. Accounting for a potential 50% loss to follow-up or refusal to participate, the final estimated sample size was adjusted to at least 398 participants per group. Finally, a total of 400 non-diarrheal subjects and 670 acute diarrheal children were recruited in the study.

### Questionnaire survey

A structured questionnaire was used to collect demographic and clinical information from both diarrheal cases and non-diarrheal children, including sex, age, place of residence, date of visit, and medical history such as lactose intolerance, systemic lupus erythematosus, Henoch–Schönlein purpura, and cow’s milk protein allergy, etc. For diarrheal cases, additional clinical data related to enteric infections were obtained, including the number of diarrheal episodes within the past 24 h, duration of diarrhea, presence of fever, vomiting, dehydration, seizures, metabolic acidosis, myocardial injury, and stool characteristics (e.g., watery, bloody, or mucoid stool).

Prior to study initiation, a standardized protocol was developed and all participating staff, including physicians and nurses, received comprehensive training to ensure effective communication with children’s caregivers or parents and standardized data collection. During the study period, strict adherence to inclusion and exclusion criteria was maintained. Data were double-entered using REDCap software (version 10.0, Vanderbilt University, Nashville, United States), and cross-validation procedures were conducted before finalizing the dataset. Each questionnaire was reviewed by quality control personnel. In cases of discrepancies or missing information, questionnaires were subject to thorough examination, and participants were promptly contacted to verify and clarify responses.

### Specimen collection

Single stool samples (> 3 g or > 3 mL) were collected from each enrolled participant using sterile sampling cups at the four hospital sites and temporarily stored at 4 ℃ [8, 9]. All samples were transported within 12 h to the central laboratory at Guangzhou Women and Children’s Medical Center using temperature-controlled transport containers maintained at 4 ℃. Upon arrival, each stool sample was divided into three aliquots for subsequent testing. One portion was used immediately for bacterial culture, including inoculation and enrichment procedures for the isolation and identification of bacterial pathogens, including DEC subtypes, NTS, *Shigella* spp., *Vibrio  cholerae,* *Vibrio parahaemolyticus* (VP), *Plesiomonas* spp, *Aeromonas*, spp. [8, 9].  A second portion (approximately 0.20 g) was stored at − 70 ℃ for ribonucleic acid (RNA) extraction and subsequent detection of enteric viruses, including RVA, NoVs, and astrovirus (As) [8, 9]. The third portion (approximately 0.20 g) was also stored at − 70 ℃ for deoxyribonucleic acid (DNA) extraction and used to detect *Cryptosporidium * spp., *Giardia lamblia*, *Entamoeba histolytica* and *Blastocystis hominis* (Bh), adenovirus (Ad), *Clostridioides difficile* (CD), *Campylobacter jejuni, Listeria monocytogenes*, and *Yersinia enterocolitica* [19–23].

### Enteric pathogens detection

A total of 21 enteric pathogens were targeted in this study, comprising 11 bacterial species, 6 enteric viruses, and 4 protozoan parasites. Each enrolled participant underwent comprehensive testing for all 21 pathogens using a panel of standardized laboratory techniques.

Bacteriological diagnostic tests included traditional culture methods combined with serological identification, biochemical assays, and quantitative real-time polymerase chain reaction (qPCR) assays (Table S1). The following diarrheagenic bacteria were tested: DEC, NTS, *Shigella* spp., *Vibrio cholerae*, VP, *Aeromonas* spp., *Plesiomonas* spp., CD, *Campylobacter jejuni* (CJ), *Listeria monocytogenes*, and *Yersinia enterocolitica*. MacConkey agar (Oxoid Ltd, Basingstoke, UK) was used for culturing DEC, which was divided into five subtypes by their virulence genetic as following: enteroaggregative *Escherichia coli* (EAEC), enterotoxigenic *Escherichia coli* (ETEC), enteropathogenic *Escherichia coli* (EPEC), enteroinvasive *Escherichia coli* (EIEC) and enterohaemorrhagic *Escherichia coli* (EHEC). The DEC subtypes were examined with qPCR based on the previous literature [8]. Each stool sample was inoculated into the selenite brilliant green sulfa enrichment broth (Oxoid Ltd, Basingstoke, UK) for enrichment and then inoculated it onto *Salmonella*-*Shigella* agar (Oxoid Ltd, Basingstoke, UK) to detect NTS. In addition, each stool specimen was inoculated directly onto *Salmonella*-*Shigella* agar (Oxoid Ltd, Basingstoke, UK) to find *Shigella* spp.. In addition, each sample was inoculated onto alkaline peptone water (Oxoid Ltd, Basingstoke, UK) for enrichment, and then inoculated onto thiosulfate-citrate-bile salts-sucrose agar (Oxoid Ltd, Basingstoke, UK) to detect *Vibrio cholera*, VP, *Aeromonas* spp. and *Plesiomonas* spp. [8]. For suspicious NTS, *Shigella* spp., *Vibrio cholera*, VP, *Aeromonas* spp., and *Plesiomonas* spp. colonies, the systematic biochemical identification of was performed using the VITEK^®^ 2 Compact instrument (bioMerieux, Marcyl’ Etoile, France). Detailed detection procedures have been described previously [8]. In addition, all stool specimes were subjected to fecal genomic DNA extraction with QIAamp DNA stool mini kit (Qiagen, Hilden, Germany) according to the manufacturers’ protocol, the qPCR was conducted to find *Listeria  monocytogenes* [21], CJ [22], CD [23] and *Yersinia  enterocolitica* [21], detailed detection procedures have been described previously [21–23].

The stool specimens obtained from enrolled children were later tested for RVA, NoVs, As, Ad nucleic acid by qPCR with commercial kits (Qiagen, Hilden, Germany. Table S1). Nucleic acid was extracted from each stool specimen (15% wt / vol or vol/vol suspension) with QIAamp Viral RNA Kit (Qiagen, Hilden, Germany). The reverse transcription (RT)-PCR was conducted with PrimeScript™ RT reagent Kit (Takara Bio Inc, Shiga, Japan) to product complementary DNA (cDNA), and then the cDNA was applied to detected RVA [22], NoVs (GI, GII) [24], Sapovirus [25], and As [26] with qPCR, respectively. Ad is a DNA virus, and its nucleic acids are directly extracted and detected through qPCR for the diagnosis of Ad infection [26].

A portion of stool was conducted to extracted the genomic DNA of Bh, *Cryptosporidium* spp., *Giardia lamblia*, and *Entamoeba histolytica* was extracted from each stool sample with QIAamp DNA stool mini kit (Qiagen, Hilden, Germany) according to the manufacturers’protocol (Table S1).The qPCR was applied to detect these four intestinal protozoa [27, 28].

Positive DNA or cDNA and negative (DNA or RNA matrix replaced by water) qPCR controls were performed in all testing. Samples with a cycle threshold (Ct) values greater than the Ct values of the positive control alone (diluted by tow folds) were subjected to repeat testing in a qPCR after a tenfold dilution.

Importantly, all laboratory personnel were blinded to the clinical status of the samples, and were unaware whether the specimens originated from diarrheal cases or non-diarrheal controls throughout the testing procedures.

### Sensitivity analysis

To assess the robustness of the primary findings regarding risk factors for diarrhea, a sensitivity analysis was performed to address the question: how strong would any unmeasured confounding need to be to negate our statistically significant associations? The first approach examined the impact of measured confounders on acute diarrhea by modifying the statistical methodology. Specifically, A least absolute shrinkage and selection operator (Lasso) logistic regression model with tenfold cross-validation was applied, using the minimum lambda criterion to optimize model performance [19, 29]. The variables selected through Lasso were then entered into a multivariable binary logistic regression model to evaluate their independent associations with acute diarrhea.

The second approach also addressed measured confounders by applying several novel artificial intelligence (AI) algorithms to construct predictive models. Specifically, the models included light gradient boosting machine (LightGBM), support vector machine (SVM), random forest (RF), classification and regression tree (CART), and eXtreme gradient boosting (XGBoost) algorithms [19]. Prior to model construction, the entire dataset was randomly divided into training and testing sets in a 7∶3 ratio. Given the limited number of variables, no feature selection was performed; all available variables were included in each model. Model performance was evaluated using multiple metrics, including the area under the receiver operating characteristic curve (AUROC), accuracy, sensitivity, specificity, recall, and F1 score [19]. Feature importance scores were generated to quantify the contribution of each predictor to acute diarrhea, with higher scores indicating stronger associations [19]. If the same high-ranking predictors consistently emerged across multiple machine learning models, this was considered evidence of a robust association between these variables and acute diarrhea.

### Additive and multiplicative interaction of co-infection effects

The pathogenicity of each enteric pathogen was quantified using odds ratios (*OR*s) based on the presence of the pathogen and the occurrence of diarrheal symptoms. These *OR*s were calculated directly from 2 × 2 contingency tables. We estimated the Mantel–Haenszel pooled *OR* for both single and co-infections to assess their associations with diarrhea [7, 30]. Additive interaction between two pathogens was assessed using the interaction contrast ratio (ICR), also known as the relative excess risk due to interaction (RERI), along with age-standardized *OR*s. The ICR was calculated using the following formula [7, 30].

$${\text{ICR}} = OR_{{{\text{co}} - {\text{infection}}}} - OR_{{\text{single infection 1}}} - OR_{{\text{single infection 2}}} + { 1}$$

The multiplicative scale, by estimating departure from multiplicativity of age-standardized *OR*s [7], which we refer to as “multiplicative interaction” (Mul):

$${\text{Mul }} = OR_{{{\text{co}} - {\text{infection}}}} / \, \left( {OR_{{\text{single infection 1}}} \times OR_{{\text{single infection 2}}} } \right)$$

To make statistical inferences while accounting for correlated data, we characterized the sampling distributions of the ICR, Mul, and all *ORs* using bootstrap resampling. For each bootstrap iteration, we sampled with replacement from the original data set to create a new dataset with the same sample size as the original. Using this resampled dataset, we calculated estimates of the ICR, Mul, and all *ORs*. This process was repeated 1000 times to produce estimates of the sample distributions associated with each statistic. The 2.5^th^ and 97.5^th^ percentiles of the resulting bootstrap distributions were used as 95% confidence intervals (*CI*s) [31].

### Data analysis

#### Statistical modeling

Missing data was imputed using a technique known as multiple imputation (20 imputed datasets), which accounts for the uncertainty in the generated values through the addition of randomness during the imputation procedure [19].

For continuous variables (e.g., number of diarrheal episodes, duration of diarrhea, and age), normality assumptions were assessed using the Kolmogorov-Smirnov test with Lilliefors correction. *P* < 0.05 was considered indicative of non-normal distribution. If variables were normally distributed and exhibited homogeneity of variance between groups (e.g., acute diarrhea vs. non-diarrhea, or between different pathogen types), results were expressed as mean ± standard deviation, and comparisons were performed using independent samples *t* tests. For non-normally distributed variables and/or those with unequal variances between groups, results were summarized as median with interquartile range (IQR: Q1, Q3), and comparisons were made using the Mann–Whitney *U* test. Categorical variables (e.g., sex, age group, and pathogen category) were presented as counts and percentages, and group comparisons were conducted using the Pearson *χ*^2^ test, Fisher’s exact test, or likelihood ratio *χ*^2^ test, as appropriate [8, 19, 20].

A binary logistic regression model was used to examine variables that were related to diarrheal disease, with gender, age, season, and the enteric pathogens included as explanatory variables. Multivariate analysis was performed by including all variables with *P* < 0.20 from the univariate analysis as covariates. The *OR*s and 95% *CI*s were estimated using maximum likelihood methods [8, 19, 20].

#### Co-occurrence of enteric pathogens

Null model analysis was employed to investigate the associations—whether positive, negative, or random—among enteric co-infections. Data were structured into a 4 × 507 presence-absence matrix, where each row represented a specific pathogen species and each column represented an individual study participant. Within this matrix, a "1" denoted the presence of a species in a host, while a "0" indicated its absence [8].

The C-score, a co-occurrence index, was employed to characterize interaction patterns among species. This study utilized a fixed row-equiprobable column algorithm for the analysis. The derived C-score was benchmarked against the expected values generated from 5000 null matrices, assembled randomly and evaluated via Monte Carlo simulations. Additionally, a standardized effect size was computed to assess the extent of co-occurrence; this metric quantifies the deviation of the observed C-score from the average of the simulated distributions, in terms of standard deviations. Analyses were conducted using the EcoSimR-package of R software (version 4.3.3; R Foundation for Statistical Computing, Vienna, Austria; available at https://cran.r-project.org), as described in the literature [8, 32, 33].

#### Assessing the impact of co-infection with enteric pathogens on diarrhea severity

The study utilized partial least squares (PLS) regression to assess the impact of enteric pathogen co-infection on clinical symptoms of diarrhea. This method was chosen for its capacity to handle complex ecological data and its robustness in the presence of multicollinearity, making it particularly suitable for exploring the nuanced effects of coinfections on health, including the impact of coinfections on the host's health [34], and its distribution is free and well suited to deal with multicollinearity [32]. In our model, the explanatory component consisted of a presence-absence matrix for 21 enteric pathogens, with age also included as a covariate to account for age-related variations in clinical presentation [8]. The response component encapsulated the primary symptoms associated with these infections, such as diarrhea in the past 24 h, fever, vomiting, dehydration, convulsion, acidosis, and anemia.

The validity of the PLS model was evaluated using Stone-Geisser's Q2 test, a cross-validation redundancy measure designed to assess the predictive relevance of exogenous variables. A Q2 value exceeding 0.0975 signifies statistical significance of the predictors, whereas values below this threshold indicate a lack of significance. Additionally, the proportion of the observed variability in mean normalized least squares explained by the enteric pathogen block was calculated. The analyses were conducted using the "plspm" package of R software (version 4.3.3; R Foundation for Statistical Computing, Vienna, Austria; available at https://cran.r-project.org) [8, 32].

All statistical analyses were performed with R software (version 4.3.3; R Foundation for Statistical Computing, Vienna, Austria; available at https://cran.r-project.org) and RStudio Desktop (version 1.2.5033; RStudio, Inc., Boston, Massachusetts, United States).

## Results

### Baseline characteristics

From January 1, 2024 to December 31, 2024, a total of 1070 children under the age of five were enrolled from four sentinel hospitals, including 670 children with acute diarrhea and 400 non-diarrheal controls. The median age of all participants was 17 months (IQR: 8, 38); the median age was significantly lower among diarrheal cases (14 months; IQR: 8, 26) compared with controls (32 months; IQR: 10, 5;  *Z* = 8.201, *P* < 0.001). Among the 1070 participants, 369 were female, accounting for 34.0% (228/670) of diarrheal cases and 35.3% (114/400) of controls, with no significant difference in sex distribution between groups (*χ*^2^ = 0.165, *P* = 0.685). A total of 869 participants were recruited from outpatient clinics, including 534 (79.7%) diarrheal cases and 325 (81.3%) controls, with no significant difference in recruitment setting between groups (*χ*^2^ = 0.379, *P* = 0.538). Regarding seasonal distribution, 91 (22.8%), 147 (36.8%), 92 (23.0%), and 70 (17.5%) diarrheal cases were enrolled in spring, summer, autumn, and winter, respectively, compared with 166 (24.8%), 216 (32.2%), 162 (24.2%), and 126 (18.8%) non-diarrheal controls. No significant seasonal variation was observed between groups (*χ*^2^ = 2.311, *P* = 0.511; Additional file 1: Table S2).

Among the 670 children with acute diarrhea, 172 (25.7%) presented with fever, 181 (27.0%) experienced vomiting, 26 (3.9%) had signs of dehydration, 5 (0.7%) had myocardial injury, 3 (0.4%) were diagnosed with metabolic acidosis, and 4 (0.6%) had anemia. In addition, 178 children (26.6%) passed watery stools. The median number of diarrheal episodes in the 24 h prior to presentation was 5 (IQR: 4, 7).

### Diarrhea-related enteric pathogens

The overall detection rate of any enteric pathogen was significantly higher among diarrheal children than non-diarrheal children (53.6% vs. 27.8%; *χ*^2^ = 67.856, *P* < 0.001). Among children with diarrhea, viral pathogens were the most commonly detected (*n* = 229, 34.2%), followed by bacterial (*n* = 156, 23.3%) and parasitic pathogens (*n* = 46, 6.9%). In contrast, among non-diarrheal children, bacterial pathogens were most prevalent (*n* = 53, 13.3%), followed by viral (*n* = 50, 12.5%) and parasitic pathogens (*n* = 19, 4.8%). The detection rates of both bacterial and viral pathogens were significantly higher in the diarrheal group than in the control group (bacterial: *χ*^2^ = 16.043, *P* < 0.001; viral: *χ*^2^ = 61.069, *P* < 0.001), whereas there was no significant difference in the detection of parasitic pathogens between the two groups (*χ*^2^ = 1.965, *P* = 0.161).

Among children with diarrhea (*n* = 670), the five most frequently detected pathogens were RVA (*n* = 177, 26.4%), NoVs GII (*n* = 73, 10.9%), DEC (*n* = 52, 7.8%), CD (* n* = 47, 7.0%), and Bh (*n* = 36, 5.4%). In non-diarrheal children (*n* = 400), the most commonly detected pathogens were RVA (*n* = 28, 7.0%), DEC (*n* = 17, 4.3%), Bh (*n* = 17, 4.3%), and NoVs (*n* = 16, 4.0%). No cases of *Vibrio cholerae*, As, or sapovirus were detected in either diarrheal or non-diarrheal children.

The detection rate of DEC was significantly higher in children with diarrhea than in non-diarrheal controls (*χ*^2^ = 5.199, *P* = 0.024). Notably, five DEC subtypes were tested in this study (Table 1), among which only EPEC and EAEC were identified. No cases of EIEC, EHEC, or ETEC were detected in either group. EPEC was significantly associated with diarrhea in children under five years of age (*χ*^2^ = 6.983, *P* = 0.008), whereas EAEC showed no statistically significant association with diarrheal status (*χ*^2^ = 0.172, *P* = 0.678).

**Table 1:** Univariate logistic regression analysis of associations between enteric pathogen infections and diarrhea in children under five years of age

Pathogens	Non-diarrhea subjects *n* = 400 *n* (%)	Acute diarrhea cases *n* = 670 *n* (%)	*χ*^2^	*P*	*OR* (95% *CI*)
Any one pathogen	111 (27.8)	359 (53.6)	67.856	< 0.001	3.005 (2.303–3.923)
Any one bacterium	53 (13.3)	156 (23.3)	16.043	< 0.001	1.987 (1.414–2.792)
Any one virus	50 (12.5)	229 (34.2)	61.069	< 0.001	3.635 (2.596–5.089)
Any one parasite	19 (4.8)	46 (6.9)	1.965	0.161	1.478 (0.853–2.561)
DEC	17 (4.3)	52 (7.8)	5.119	0.024	1.896 (1.080–3.326)
EPEC	9 (2.3)	38 (5.7)	6.983	0.008	2.612 (1.249–5.461)
EAEC	8 (2.0)	16 (2.4)	0.172	0.678	1.199 (0.508–2.827)
EIEC	0 (0.0)	0 (0.0)	–	–	–
ETEC	0 (0.0)	0 (0.0)	–	–	–
EHEC	0 (0.0)	0 (0.0)	–	–	–
Non-typhoidal *Salmonella*^a^	3 (0.8)	10 (1.5)	1.151	0.283	2.005 (0.549–7.329)
*Shigella* ^a^	1 (0.3)	8 (1.2)	2.676	0.102	4.822 (0.601–38.695)
*Vibrio cholerae*	0 (0.0)	0 (0.0)	–	–	–
*Vibrio parahaemolyticus*	3 (0.8)	20 (3.0)	5.949	0.015	4.072 (1.202–13.790)
*Plesiomonas*	2 (0.5)	7 (1.0)	0.891	0.345	2.101 (0.434–10.164)
*Aeromonas*	8 (2.0)	8 (1.2)	1.105	0.293	0.592 (0.220–1.590)
*Yersinia enterocolitica *^*a*^	6 (1.5)	6 (0.9)	0.825	0.364	0.593 (0.190–1.852)
*Listeria monocytogenes *^*a*^	1 (0.3)	2 (0.3)	0.021	0.885	1.195 (0.108–13.217)
*Campylobacter jejuni*	5 (1.3)	15 (2.2)	1.335	0.248	1.809 (0.653–5.016)
*Clostridioides difficile*	10 (2.5)	47 (7.0)	10.124	0.001	2.942 (1.470–5.891)
Group A rotavirus	28 (7.0)	177 (26.4)	60.975	< 0.001	4.770 (3.132–7.265)
Norovirus GII	16 (4.0)	73 (10.9)	15.617	< 0.001	2.935 (1.683–5.117)
Norovirus GII^a^	1 (0.3)	1 (0.1)	0.136	0.716	0.596 (0.037–9.562)
Sapovirus	0 (0.0)	0 (0.0)	–	–	–
Adenovirus	9 (2.3)	19 (2.8)	0.337	0.581	1.268 (0.568–2.830)
Astrovirus	0 (0.0)	0 (0.0)	–	–	–
*Blastocystis hominis*	17 (4.3)	36 (5.4)	0.671	0.413	1.279 (0.709–2.309)
*Entamoeba histolytica *^*a*^	1 (0.3)	1 (0.1)	0.136	0.712	0.596 (0.037–9.562)
*Giardia lamblia *^*a*^	2 (0.5)	8 (1.2)	1.303	0.254	2.405 (0.508–11.381)
*Cryptosporidium *^*a*^	1 (0.3)	4 (0.6)	0.648	0.421	2.396 (0.267–21.516)

Notes: In the calculations for this table, con-infections of enteric pathogen were not considered.

"a" represents the Fisher exact test.

"–" represents unavailability of calculation.

*CI* Confidence interval, *DEC* diarrheagenic *Escherichia coli,* *EAEC* Enteroaggregative *Escherichia coli, EPEC* Enteropathogenic *Escherichia coli,* *EHEC* Enterohemorrhage *Escherichia coli,* *EIEC* Enteroinvasive *Escherichia coli,* *ETEC* Enterotoxigenic *Escherichia coli, OR Odds ratio*

RVA, NoVs GII, CD, and VP were detected significantly more frequently in children with diarrhea than in non-diarrheal controls (*χ*^2^ = 60.975, *P* < 0.001; *χ*^2^ = 15.617, *P* < 0.001; *χ*^2^ = 10.124, *P* = 0.001; *χ*^2^ = 5.949, *P* = 0.015, respectively). In contrast, no statistically significant differences in detection rates were observed between groups for other pathogens, including NTS, *Shigella* spp., *Plesiomonas* spp., *Aeromonas* spp., *Yersinia enterocolitica*, *Listeria monocytogenes*, CJ, NoVs GI, Ad, Bh, *Entamoeba histolytica*, *Giardia  lamblia*, and *Cryptosporidium * spp. (Table 1).

A multivariable logistic regression analysis was conducted including seven candidate variables: age (months), RVA, NoVs GII, DEC, CD, VP, and *Shigella* spp. Variable selection was performed using a stepwise approach. The final model identified five pathogens significantly associated with diarrhea: DEC (*OR* = 1.927; 95% *CI:* 1.031–3.368), VP (*OR* = 6.221; 95% *CI:* 1.738–22.267), CD (*OR* = 2.482; 95% *CI:* 1.185–5.201), RVA (*OR* = 4.487; 95% *CI:* 2.902–6.941), and NoVs GII (*OR* = 2.103; 95% *CI:* 1.160–3.812). Younger age was also independently associated with an increased risk of diarrhea (Table 2). The AUROC for the final model was 0.733 (95% *CI:* 0.701–0.764), indicating moderate discriminatory performance (Additional file 1: Table S3).

**Table 2:** Multivariate logistic regression analysis of enteric pathogens associated with diarrhea in children under five years

Variables	B	S.E.	Wald	df	*P*	*OR* (95% *CI*)
Age (months)	− 0.036	0.004	86.044	1	< 0.001	0.965 (0.958–0.972)
DEC	0.603	0.309	3.796	1	0.051	1.927 (1.032–3.368)
*Vibrio parahaemolyticus*	1.828	0.651	7.892	1	0.005	6.221 (1.738–22.267)
*Clostridioides difficile*	0.909	0.377	5.804	1	0.016	2.482 (1.185–5.201)
Group A rotavirus	1.501	0.223	45.502	1	< 0.001	4.487 (2.902–6.941)
Norovirus GII	0.743	0.304	5.994	1	0.014	2.103 (1.160–3.812)
Constant	1.004	0.122	67.670	1	< 0.001	–

All reported* P* values were not adjusted for multiple comparisons using the Bonferroni correction method.

‘–’ indicates that data could not be calculated.

*CI*s Confidence intervals, *DEC* Diarrheagenic *Escherichia coli, OR* Odds ratio, S.E. Standard error, *df* Degrees of freedom

### Sensitivity analysis

Lasso logistic regression identified several key pathogens associated with diarrhea, including RVA, VP, DEC, NTS, NoVs GII, and CD (Additional file 1: Figure S1A–C). To further validate these findings, multiple machine learning models—including CART, RF, XGBoost, SVM, and LightGBM—were employed to systematically assess the determinants of pediatric diarrhea. Among these, XGBoost demonstrated the best predictive performance, with an AUROC of 0.795 (95% *CI*: 0.762–0.827) in the training set and a robust AUROC of 0.754 (95% *CI*: 0.697, 0.827) and accuracy of 0.901 in the testing set. Across all models, age and RVA were consistently identified as the most influential predictors, while DEC, CD, NoVs GII, and Bh also exhibited high importance in selected algorithms (Additional file 1: Table S4).

### Co-infections of enteric pathogens

Co-infections involving multiple enteric pathogens were commonly observed in both diarrheal and non-diarrheal children, with a significantly higher prevalence in the diarrheal group (16.1% vs. 4.5%, *χ*^2^ = 32.592, *P* < 0.001). Among the 21 pathogens tested in this study, a wide array of co-infection patterns was identified. In diarrheal cases, co-infections involving two, three, four, and even five pathogens were detected, whereas in non-diarrheal children, only dual and triple pathogen co-infections were observed. The diversity and complexity of co-infection combinations were significantly greater in diarrheal children than in non-diarrheal controls (*χ*^2^ = 74.852, *P* < 0.001). Specifically, dual-pathogen co-infections were more prevalent in diarrheal children (13.0% vs. 4.0%, *χ*^2^ = 23.234, *P* < 0.001), as were triple-pathogen co-infections (2.4% vs. 0.5%, *χ*^2^ = 5.398, *P* = 0.020). No significant differences were found between groups for co-infections involving four or five pathogens. Among children with co-infections, dual-pathogen combinations accounted for the majority in both groups—80.5% (87/108) among diarrheal children and 88.9% (16/18) among non-diarrheal children (Table 3).

**Table 3:** Co-infections among diarrheal and non-diarrheal children under five years of age

Variables	Non-diarrhea subjects *n* = 400 *n* (%)	Acute diarrhea cases *n* = 670 *n* (%)	*χ*^2^	*P*	*OR* (95%* CI*)
DEC-group A rotavirus ^a^	1 (0.3)	12 (1.8)	4.956	0.026	7.277 (0.943–56.174)
*Clostridioides difficile*-group A rotavirus	0 (0.0)	17 (2.5)	10.313	< 0.001	–
Group A rotavirus-Norovirus GII	2 (0.5)	33 (4.9)	15.503	< 0.001	10.309 (2.460–43.199)
DEC-*Blastocystis hominis*^a^	0 (0.0)	7 (1.0)	4.207	0.041	–
Group A rotavirus-adenovirus^a^	2 (0.5)	8 (1.2)	1.303	0.254	2.405 (0.508–11.381)
DEC-Norovirus GII^a^	1 (0.3)	8 (1.2)	2.676	0.102	4.822 (0.601–38.695)

In this analysis, we only reported co-infection combinations whose proportion exceeded 1% among the study population.

"a" represents the Fisher exact test.

"–" represents unavailability of calculation.

*CI*s Confidence intervals, *OR* Odds ratio, *DEC* Diarrheagenic *Escherichia coli*

Among children with diarrhea, the most common type of co-infection involved virus-virus combinations (*n* = 39, 5.8%), followed by bacteria-parasite (*n* = 18, 2.7%) and bacteria-bacteria co-infections (*n* = 16, 2.4%). In contrast, among non-diarrheal children, virus–virus co-infections remained the most frequent (*n* = 4, 1.0%), followed by bacteria-parasite (*n* = 4, 1.0%) and bacteria–bacteria co-infections (*n* = 2, 0.5%). Virus-virus (*χ*^2^ = 15.095, *P* < 0.001) and bacteria-bacteria (*χ*^2^ = 5.399, *P* = 0.021) co-infections occurred significantly more often in diarrheal children compared to non-diarrheal children (Table S5).

Further analysis of dual-pathogen combinations revealed that RVA-NoVs GII was the most prevalent co-infection in diarrheal children (*n* = 33, 4.9%), followed by RVA-CD (*n* = 17, 2.5%), RVA-DEC (*n* = 12, 1.8%), RVA-Ad (*n* = 5, 1.2%), and DEC-NoVs GII (*n* = 8, 1.2%). In contrast, the frequency of all dual-pathogen co-infection combinations in non-diarrheal children remained below 1.0%, with RVA-NoVs GII and RVA-Ad each detected in two cases (0.5%). Statistically significant differences were observed in the prevalence of RVA-DEC (*χ*^2^ = 4.956, *P* = 0.026), RVA-CD (*χ*^2^ = 10.313, *P* < 0.001), RVA-NoVs GII (*χ*^2^ = 15.503, *P* < 0.001), and DEC-Bh (*χ*^2^ = 4.207, *P* = 0.041), all of which were more frequently detected in children with diarrhea compared to those without (Table 3). No significant differences were found for other co-infection combinations between the two groups (Additional file 1: Table S6).

### Enrichment effects of pathogens on diarrheal severity

**Fig. 1:**
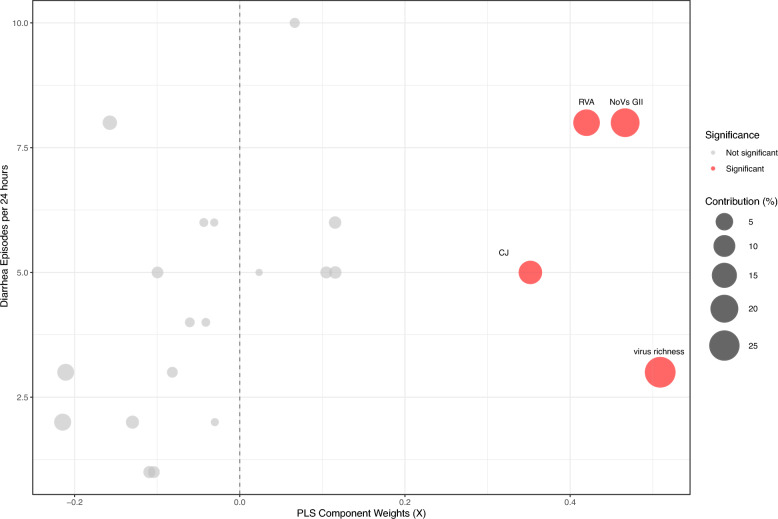
Association between PLS-derived pathogen weights and diarrhea frequency, with color indicating significance (red: significant; grey: non-significant). *CJ* *Campylobacte jejuni*, *CI*s Confidence intervals, *DEC* Diarrheagenic *Escherichia coli,* *NoVs GII* Norovirus GII, *RVA* Group A rotavirus, *PLS* Partial least squares

PLS regression analysis identified virus richness, NoVs GII, RVA, and CJ as the principal contributors to diarrheal severity in children, with respective contribution percentages of 25.907%, 21.770%, 17.626%, and 12.378%, all exceeding the threshold of 5%. The weights for these variables were all positive, indicating that virus richness, RVA, and NoVs GII were not only highly influential factors (high weight scores) but were also positively correlated with the number of diarrheal episodes. These findings suggest that co-infection involving these pathogens may substantially exacerbate diarrheal symptoms (Table 4. Figure 1).

**Table 4:** Predictor weights of the PLS model explaining the severity of acute diarrhea in children aged under five years

Pathogen	Loading	Weight	Percent contribution	Correlation	*P*
Virus richness	0.509	0.509	25.907	0.160	< 0.001
Norovirus GII	0.467	0.467	21.770	0.147	< 0.001
Group A rotavirus	0.420	0.420	17.626	0.132	0.001
*Campylobacter jejuni*	0.352	0.352	12.379	0.111	0.004
*Clostridioides difficile*	− 0.214	− 0.214	4.598	− 0.068	0.081
Non-typhoidal *Salmonella*	− 0.211	− 0.211	4.443	− 0.066	0.086
Diarrheagenic* Escherichia coli*	− 0.130	− 0.130	1.689	− 0.041	0.290
*Yersinia enterocolitica*	0.116	0.116	1.336	0.036	0.347
*Shigella*	0.115	0.115	1.331	0.036	0.348
Adenovirus	− 0.109	− 0.109	1.198	− 0.034	0.373
*Blastocystis hominis*	0.105	0.105	1.096	0.033	0.394
*Giardia lamblia*	− 0.104	− 0.104	1.084	− 0.033	0.397
Bacteria richness	− 0.100	− 0.100	0.992	− 0.031	0.417
Norovirus GI	− 0.082	− 0.082	0.667	−0.026	0.506
*Listeria monocytogenes*	0.067	0.067	0.443	0.021	0.588
*Cryptosporidium*	− 0.061	− 0.061	0.366	− 0.019	0.622
*Vibrio parahaemolyticus*	− 0.044	− 0.044	0.190	− 0.014	0.723
*Plesiomonas*	− 0.041	− 0.041	0.169	− 0.013	0.738
*Aeromonas*	− 0.031	− 0.031	0.096	− 0.010	0.801
*Entamoeba histolytica*	− 0.030	− 0.030	0.091	− 0.010	0.806
Protozoa richness	0.023	0.023	0.055	0.007	0.849

Predictor weights represent the contribution of pathogen to the PLS X component. Cross-correlations represent the correlations between each pathogen and the number of diarrheal episodes in the past 24 h (as an indicator of diarrhea severity). Virus, protozoa, and bacteria richness signify the maximum number of species from each group detected in a child.

*PLS* Partial least squares

### Interaction patterns of enteric pathogen co-infections

**Fig. 2:**
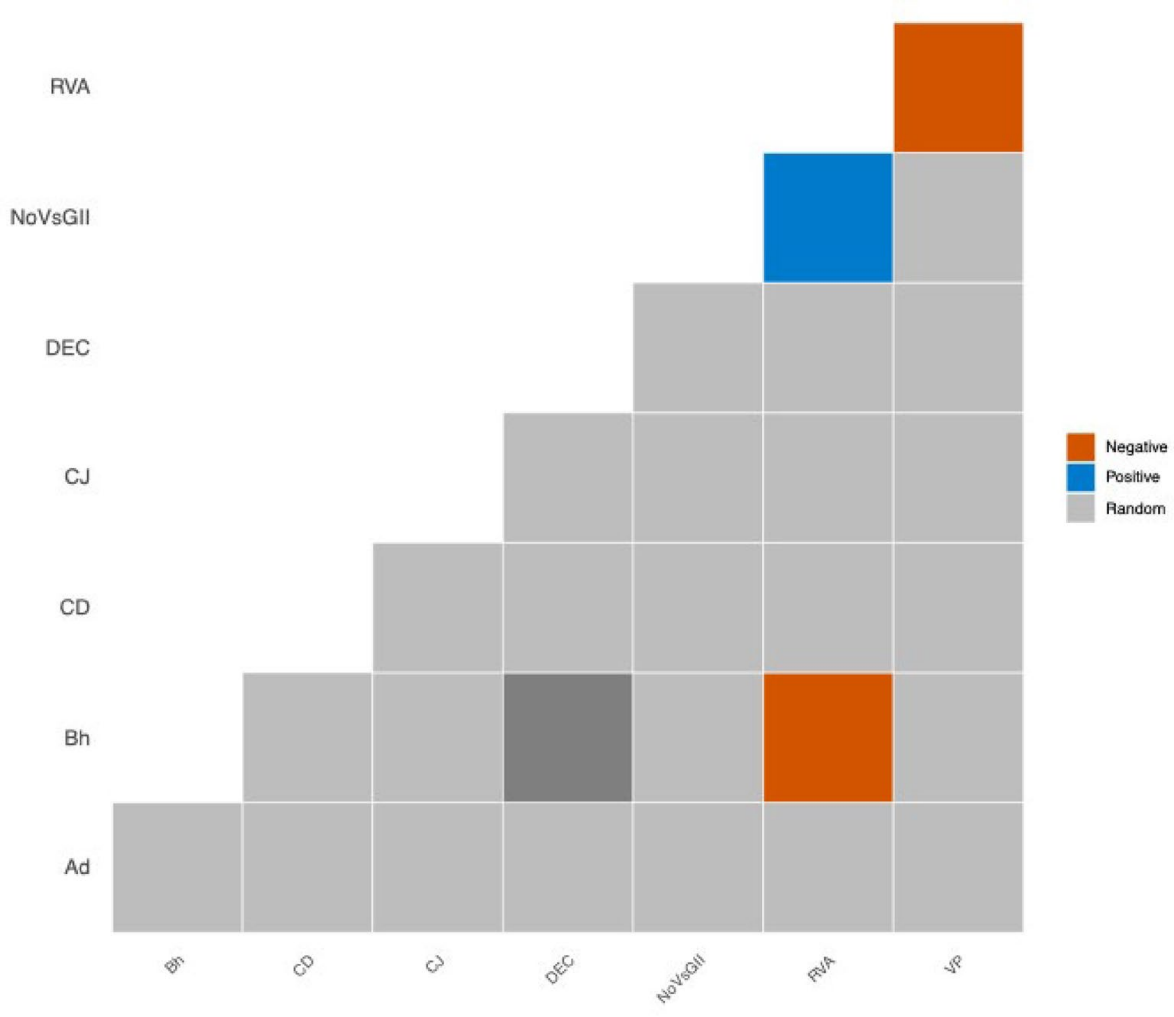
Heatmap showing associations between significant enteric pathogen species determined using the probabilistic co-occurrence model for the enteric pathogens detected in 670 children (< 5 years) suffering from acute diarrhea. The column and row represent pairwise relationship between two enteric pathogens. Boxes in grey color indicate random co-occurrences, and orange boxes indicate associations were less common than expected by chance. *Ad* Adenovirus, *CD* *Clostridioides difficile*, *CJ* *Campylobacter jejuni*, *DEC* Diarrheagenic *Escherichia coli*, *Bh* *Blastocystis hominis*, *NoVs GII* Norovirus GII, *RVA* Group A rotavirus, *VP* *Vibrio parahaemolyticus*

The study evaluated pairwise co-infection patterns among eight prevalent enteric pathogens in children with diarrhea, aiming to identify potential non-random interactions by comparing observed co-infection frequencies with expected values under an assumption of independent distribution. A notably higher-than-expected co-occurrence was identified for RVA and NoVs GII (33 observed vs. 19.3 expected; p_gt < 0.001), as well as for DEC and Bh (7 observed vs. 2.8 expected; p_gt = 0.016), suggesting possible synergistic interactions between these pathogen pairs. In contrast, RVA-Bh co-infection was observed in only one case, substantially below the expected frequency of 9.5 (p_lt < 0.001), and RVA-VP co-infection also occurred less frequently than anticipated (p_lt = 0.017), indicating statistically significant negative deviations suggestive of antagonistic or mutually exclusive relationships within the host (Fig. 2, Table 5).

**Table 5:** Pairwise probability table showing single infections and co-infections with enteric pathogens in diarrheal children aged under five years

Species 1	Species 2	Species 1 name	Species 2 name	Species 1 infection	Species 2 infection	Co-infection	Probability occurrence	Expected occurrence	p_lt	p_gt
1	2	Group A rotavirus	Norovirus GII	177	73	33	0.029	19.3	1.000	0.000
1	3	Group A rotavirus	DEC	177	52	12	0.021	13.7	0.350	0.765
1	4	Group A rotavirus	*Clostridioides difficile*	177	47	17	0.019	12.4	0.956	0.083
1	5	Group A rotavirus	*Blastocystis hominis*	177	36	1	0.014	9.5	0.000	1.000
1	6	Group A rotavirus	*Vibrio parahaemolyticus*	177	20	1	0.008	5.3	0.017	0.998
1	7	Group A rotavirus	Adenovirus	177	19	8	0.007	5.0	0.962	0.099
1	8	Group A rotavirus	*Campylobacter jejuni*	177	15	2	0.006	4.0	0.197	0.938
2	3	Norovirus GII	DEC	73	52	8	0.008	5.7	0.901	0.193
2	4	Norovirus GII	*Clostridioides difficile*	73	47	6	0.008	5.1	0.758	0.406
2	5	Norovirus GII	*Blastocystishominis*	73	36	3	0.006	3.9	0.433	0.775
2	6	Norovirus GII	*Vibrioparahaemolyticus*	73	20	1	0.003	2.2	0.339	0.904
2	7	Norovirus GII	Adenovirus	73	19	2	0.003	2.1	0.657	0.633
2	8	Norovirus GII	*Campylobacter jejuni*	73	15	2	0.002	1.6	0.782	0.500
3	4	DEC	*Clostridioides difficile*	52	47	5	0.005	3.6	0.853	0.297
3	5	DEC	*Blastocystis hominis*	52	36	7	0.004	2.8	0.996	0.016
3	6	DEC	*Vibrio parahaemolyticus*	52	20	3	0.002	1.6	0.938	0.197
3	7	DEC	Adenovirus	52	19	2	0.002	1.5	0.823	0.442
3	8	DEC	*Campylobacter jejuni*	52	15	2	0.002	1.2	0.897	0.327
4	5	*Clostridioides difficile*	*Blastocystis hominis*	47	36	3	0.004	2.5	0.759	0.471
4	6	*Clostridioides difficile*	*Vibrio parahaemolyticus*	47	20	0	0.002	1.4	0.228	1.000
4	7	*Clostridioides difficile*	Adenovirus	47	19	1	0.002	1.3	0.610	0.754
4	8	*Clostridioides difficile*	*Campylobacter jejuni*	47	15	0	0.002	1.1	0.332	1.000
5	6	*Blastocystis hominis*	*Vibrio parahaemolyticus*	36	20	2	0.002	1.1	0.914	0.292
5	7	*Blastocystis hominis*	Adenovirus	36	19	1	0.002	1.0	0.728	0.655

In this analysis, only children with more than five liquid stools within 24 h were considered. ‘Species 1 infection’ indicates the number of children infected with one of the species shown in the first column. The same rationale applies for ‘Species 2 infection’. The ‘%’ indicates the percentage of children co-infected by a particular pair of pathogens. Probability occurrence is the probability of a child suffering from a particular co-infection. The expected number of co-infected children is the theoretical number of children with co-infections. P_Lt and P_Gt represent the probabilities that those species could co-occur less (p_lt) than or greater (p_gt) than what is observed in our data, respectively. They can be interpreted as *P*-values, thus indicating significance levels for negative and positive co-occurrence patterns. Abbreviation: *DEC* Diarrheagenic *Escherichia coli*

### Pathogenicity and co-infections

There was a significant association between RVA single-infection and diarrhea (*χ*^2^ = 27.607, *P* < 0.001). In contrast, mono-infections with DEC (*χ*^2^ = 0.036, *P* = 0.849), NoVs GII (*χ*^2^ = 1.175, *P* = 0.185), CD (*χ*^2^ = 1.608, *P* = 0.205), and Bh (*χ*^2^ = 0.004, *P* = 0.951) were not significantly associated with diarrhea. No significant associations were observed for any other single-pathogen infections (Additional file 1: Tables S5). Co-infection of RVA and NoVs GII demonstrated a significant additive effect (ICR = 6.296, 95% *CI:* 2.061–10.723), but not a multiplicative effect (Mul = 1.865, 95% *CI*: 0.966–4.119). Co-infection with RVA and DEC exhibited both additive (ICR = 2.966, 95% *CI:* 0.851–7.248) and multiplicative effects (Mul = 2.304, 95% *CI:* 1.194–5.089), with the latter taking precedence in classification. RVA and CD co-infection showed an additive effect (ICR = 6.199, 95% *CI:* 1.701–10.601) without evidence of a multiplicative effect (Mul = 1.698, 95% *CI:* 0.821–3.652). Similarly, RVA and Bh co-infection exhibited both additive (ICR = 3.304, 95% *CI:* 1.422–5.088) and multiplicative effects (Mul = 4.602, 95% *CI:* 2.213–9.878), and was therefore classified as having a multiplicative interaction (Table 6).

**Table 6:** Assessment of the biological interactions between co-infecting pathogens associated with childhood diarrhea on additive and multiplicative scales

Infection category	*OR* (95% *CI*)	Additive model	Multiplicative model
		Interaction contrast ratio (95% *CI*)	Multiplicative interaction (95% *CI*)
Group A rotavirus (single infection)	3.375 (2.097–5.432)	–	–
Norovirus GII (single infection)	1.638 (0.784–3.420)	–	–
Group A rotavirus-Norovirus GII (co-infection)	10.309 (2.460–43.199)	6.296(2.061–10.723)	1.865 (0.966–4.119)
Group A rotavirus (single infection)	3.375 (2.097–5.432)	–	–
DEC (single infection)	0.936 (0.473–1.851)	–	–
Group A rotavirus-DEC (co-infection)	7.277 (0.943–56.174)	2.966(0.851–7.248)	2.304 (1.194–5.089)
Group A rotavirus (single infection)	3.375 (2.097–5.432)	–	–
*Clostridioides difficile* (single infection)	1.813 (0.714–4.606)	–	–
Group A rotavirus-*Clostridioides difficile* (co-infection)	10.387 (1.377–78.353)	6.199(1.701–10.601)	1.698 (0.821–3.652)
DEC (single infection)	0.936 (0.473–1.851)	–	–
*Blastocystis hominis* (single infection)	0.976 (0.456–2.089)	–	–
DEC-*Blastocystis hominis* (co-infection)	4.213 (0.516–34.366)	3.304(1.422–5.088)	4.602 (2.213–9.878)

‘-’ indicates that data could not be calculated. Group A rotavirus–*Clostridioides difficile* and DEC–*Blastocystis hominis* co-infections were each detected only once among non-diarrheal children. In this study, co-infection was defined strictly as the simultaneous presence of two pathogens; combinations involving three or more pathogens were excluded from the analysis. An additive interaction was considered absent if the 95% *CI* of the *OR* for the additive model included 0. Similarly, a multiplicative interaction was considered absent if the 95% *CI* of the *OR* for the multiplicative model included 1. When both additive and multiplicative interactions were statistically significant for a given pathogen pair, the interaction was classified as multiplicative. *CI*s Confidence intervals, *OR* Odds ratio, *DEC* Diarrheagenic *Escherichia coli*

## Discussion

This study provides a comprehensive assessment of the pathogenicity of both mono-infections and co-infections among children, with a particular focus on the synergistic interactions between enteric pathogens. Based on a hospital-based case-control design, the findings offer robust evidence that co-infecting pathogens act synergistically with RVA to increase the likelihood and severity of diarrheal illness. RVA was found to affect approximately one-fifth of the pediatric population in the study area, contributing substantially to the burden of co-infections. The observed synergistic interactions between RVA and other enteric pathogens carry important implications for global diarrhea prevention and control strategies, underscoring the urgent need for effective and widely administered RVA vaccination in infants and young children to reduce the associated morbidity and mortality.

The study identified a pathogen detection rate of 53.6% among children with diarrhea, with the most frequently detected pathogens being RVA, NoVs GII, DEC, CD, and Bh. A nationwide surveillance study in China similarly highlighted RVA, NoVs GII, DEC, Ad, and NTS as the predominant pathogens associated with pediatric diarrhea [12]. In contrast, studies conducted in African regions have reported substantially higher overall pathogen detection rates, with the most commonly identified pathogens including *Giardia lamblia*, *Shigella *spp., CJ, and *Cryptosporidium* [35]. These findings underscore marked regional differences in the etiological profile of diarrheal diseases, which may reflect underlying variations in the spatial dynamics and seasonal activity of enteric pathogens. Climatic factors—particularly temperature—play a role in driving the epidemic dynamics and seasonal peaks of diarrheal viruses, with patterns differing by latitude and longitude [12]. Moreover, the diversity of pathogens and the substantial interregional differences observed are closely linked to a combination of natural geographical conditions, levels of socioeconomic development, lifestyle factors such as dietary habits and hand hygiene, and the cultural practices of different populations [36].

The study revealed that children under the age of three exhibited a higher frequency and diversity of viral infections. Among different age groups, the peak detection rates of each viral pathogen varied. Specifically, RVA maintained a high prevalence among children aged 0–3 years, followed by a marked decline after age three. NoVs GII, on the other hand, peaked around the age of two, indicating a critical inflection point in age-specific pathogen dynamics (Additional file 1: Tables S5). In China, the age of three typically marks the beginning of preschool enrollment, which may contribute to the development of protective immunity and the subsequent reduction in infection rates. Consistent with previous findings, RVA remained the predominant viral pathogen across young children [12, 37]. NoVs GII, however, showed persistently high detection rates across all age groups, possibly due to its capacity for continual mutation and recombination, which facilitates the emergence of new strains with high epidemic potential. Unlike viral pathogens, bacterial pathogens did not demonstrate a distinct age-related pattern. The age-related variation in pathogen distribution may reflect immunological maturation or age-dependent changes in dietary habits, as well as regional epidemiological patterns, given that temperature and humidity can differentially influence the transmission of diarrheal pathogens. These age-stratified findings provide valuable evidence for optimizing the timing of pathogen-specific prevention strategies. For example, RVA vaccination should be completed within the first year of life with a booster dose administered before the age of five, while NoVs GII prevention—including vaccination once available—should be prioritized for children around the age of three.

Globally, the widespread introduction and administration of RVA vaccines have led to a substantial decline in diarrheal disease incidence. For instance, in post-vaccine era in Nicaragua, RVA infections have become rare, while other pathogens such as NoVs have become more prominent. However, despite efforts to promote RVA vaccination in China, the overall incidence of RVA gastroenteritis has not significantly declined, and RVA remains the leading cause of severe dehydrating diarrhea among children [38]. Over time, only a gradual decrease in incidence has been observed [12], suggesting suboptimal vaccine effectiveness in these settings. A major contributor to this apparent discrepancy is the high prevalence of co-infections, which complicate the accurate attribution of diarrheal disease to specific pathogens. When co-infections are appropriately accounted for in pathogen-attributable fraction analyses, the estimated effectiveness of RVA vaccines increases by 49.3% to 60.6% compared with analyses that ignore co-infection [39]. Similarly, a recent study from Botswana evaluating the Rotarix vaccine found that adjusting for co-infections with five enteric pathogens resulted in an estimated increase in vaccine effectiveness from 8.0% to 14.0% [40]. These findings highlight the critical importance of incorporating co-infection considerations into the design and analysis of future vaccine efficacy studies to achieve more accurate and reliable assessments of vaccine performance.

The study monitored that the co-infection rate among diarrheal children reached 16.1%, consistent with the 10%–17% range reported in previous studies [41–44]. The composition of co-infections is influenced not only by the population-level prevalence of individual pathogens but also by specific interactions between one pathogen (or its metabolites) and other microbes. Notably, this study identified positive associations between RVA and other enteric pathogens such as NoVs GII and CD, indicating that the presence of RVA may elevate the diarrheal risk conferred by these pathogens. This may be attributable to shared transmission routes or overlapping susceptibility profiles. Such findings are corroborated by earlier studies; for instance, one case-control study demonstrated that individuals co-infected with RVA and Giardia lamblia had a greater risk of diarrhea compared to those infected with a single pathogen [7]. Evidence from European cohorts similarly showed that coinfections were associated with more severe clinical presentations, including a higher likelihood of severe dehydration [45]. Furthermore, Chinese research has shown that the prevalence of viral, bacterial, and viral-bacterial co-infections was consistently higher in children with febrile diarrhea than in those without fever [46]. Global multicenter diarrheal studies have also reported a positive association between *Giardia lamblia* and CJ detection in both moderate and severe diarrheal cases.

The potential mechanisms underlying the synergistic effects observed in co-infections involving RVA and other enteric pathogens (e.g., DEC, etc.) may be both pathogen-specific and nonspecific. Specific mechanisms include enhanced adhesion and invasion of the intestinal epithelium by co-infecting pathogens, while nonspecific mechanisms are more likely related to host-mediated inflammatory responses. The biological pathways driving synergy may not be as straightforward as those suggested by in vitro models. RVA-induced inflammation can damage epithelial integrity and alter mucosal architecture, thereby facilitating the adherence and invasion of secondary pathogens. Inflammatory processes also lead to the release of fluids, mucins, and cellular debris, which may serve as nutrient-rich substrates for co-infecting organisms. Furthermore, the secretion of antimicrobial peptides during inflammation may disrupt the gut microbiota, enabling pathogenic species to occupy ecological niches typically filled by commensals. Collectively, these host pathological responses may foster enhanced interactions between RVA and other enteric pathogens, contributing to more severe disease outcomes.

However, antagonistic interactions—manifested as negative correlations—have been observed between certain co-infecting enteric pathogens. For instance, a negative association has been reported between RVA and *Giardia  lamblia* [35], and evidence suggests that infants co-infected with RVA and *Giardia  lamblia* may experience reduced diarrheal severity compared to those infected with RVA alone [47]. One possible mechanism underlying this protective effect is the suppression of co-infecting pathogen pathogenicity through immune-mediated or metabolic interference. Specifically, one pathogen may inhibit the replication or proliferation of another by modulating host immune responses or secreting inhibitory metabolic proteins [11, 12]. In addition, colonization or infection by one enteric pathogen may create an intestinal environment that is unfavorable to subsequent colonization by others, potentially through the induction of antimicrobial peptides, nitric oxide, reactive oxygen species, mucins, and other immune effector molecules [48].

Under the One Health framework, emerging strategies for the prevention and control of children diarrhea should adopt a holistic approach that integrates human health, animal health, and environmental factors. This approach emphasizes interdisciplinary collaboration and intersectoral coordination to establish a systematic and sustainable intervention framework. The majority of diarrheal pathogens—such as NTS and DEC—are foodborne and zoonotic in nature, with complex transmission pathways involving the food chain, water systems, animal reservoirs, and human hosts [49, 50]. Hence, First, efforts should focus on enhancing source control by promoting antibiotic-free livestock farming, controlling animal diseases, and implementing standardized microbiological management across the entire food production chain to prevent the transmission of enteric pathogens. Second, prioritizing investment in environmental sanitation and safe drinking water infrastructure is essential, along with improvements in irrigation, water source protection, and wastewater treatment capacity, to reduce children's exposure to environmental enteric pathogens [51]. In addition, a comprehensive monitoring and early warning system should be established to address the human-animal-environment interface, utilizing molecular epidemiology and genomic tracking tools to precisely identify transmission pathways of enteric pathogens and guide the development of targeted risk control strategies [52]. Furthermore, enhancing RVA vaccination coverage and advancing the development of enteric pathogen vaccines through innovative technologies can significantly reduce the health risks of diarrhea in children [53].

This study has several limitations. First, it was conducted exclusively in an urban setting, which may limit the generalizability of the findings and potentially underestimate the range of enteric pathogens circulating in broader, rural populations. Second, the exclusion of individuals with chronic diarrhea and those older than five years narrows the applicability of the results to a specific pediatric age group. Third, diarrhea severity was classified based on stool frequency within a 24-h period, with moderate diarrhea defined as more than four episodes and severe diarrhea as more than six. This operational definition does not fully align with standard grading criteria for diarrheal illness severity, potentially limiting the comparability and generalizability of the findings [54]. Fourth, although 21 enteric pathogens were tested, undetected or novel pathogens may have contributed to the diarrheal episodes. For example, the absence of stool microscopy for detecting helminths and the exclusion of other intestinal protozoa species not covered by multiplex PCR reflect limitations in the diagnostic scope. Fifth, the study was conducted in a single metropolitan area, further restricting its external validity. Sixth, the selection of sentinel hospitals was based on practical considerations, including high patient volume, diagnostic capabilities, clinical and laboratory collaborations, and regional representativeness, rather than being random. This approach may introduce selection biases. Finally, PCR primarily improves the accuracy of detection by reducing false negatives and correcting biases, thereby bringing the *OR* value closer to the true strength of the association. For highly pathogenic and difficult-to-culture pathogens, the *OR* value typically increases, while for common, weakly pathogenic or commensal microorganisms in healthy populations, the *OR* value may decrease. The core finding of the Global Enteric Multicenter Study is that molecular detection does not merely alter the size of the *OR* value, but enhances the detection accuracy, making the association strength more reflective of the true pathogenicity of the pathogen. The specific change in the *OR* value depends on the type of pathogen detected and the characteristics of the study population. However, our study did not conduct a comparison of results between different detection methods in advance [55]. Future studies should adopt large-scale, prospective, multicenter cohort designs across diverse geographic and ecological settings to comprehensively investigate the burden and interactions of mono- and co-infections in childhood diarrhea.

## Conclusions

The study comprehensively evaluated the epidemiological and etiological characteristics of acute diarrhea among children under five years of age in China. RVA and NoVs GII emerged as the predominant diarrhea-associated pathogens. Notably, co-infections of enteric pathogens demonstrated synergistic interactions, substantially increasing the risk of diarrhea and exacerbating clinical severity. These findings underscore the critical importance of early pathogen identification, reinforce the need to strengthen RVA vaccination strategies, and advocate for the development of targeted interventions based on region-specific pathogen profiles.

## Supplementary Information

Additional file 1. Table S1 All 21 enteric pathogens were tested for each individual participant using a standardized panel of laboratory techniques and diagnostic methods. Table S2. Comparison of sex, age, source, and season between diarrheal and non-diarrheal children under 5 years of age. Table S3. Model comparison of risk factors for diarrhea in children under 5 years of age based on logistic regression. Figure S1. Identification of factors associated with diarrhea in children under 5 years of age using Lasso regression. Table S4. Identification of diarrhea-associated enteric pathogens using machine learning. Table S5. Composition and comparison of enteric pathogens between diarrheal and non-diarrheal children under 5 years of age. Table S6. Comparison of single and co-infections of enteric pathogens between diarrheal and non-diarrheal children under 5 years of age.

## Data Availability

The data is confidential. To gain access, data requesters will need to sign a data access agreement, upon reasonable request. Proposals should be directed to the first author on reasonable request.

## Abbreviations

AI: Artificial intelligence
Ad: Adenovirus
As: Astrovirus
AUROC: Area under the receiver operating characteristic curve
Bh: *Blastocystis* *hominis*
CART: Classification and regression tree
CD: *Clostridioides* *difficile*
cDNA: Complementary deoxyribonucleic acid
CJ: Campylobacter jejuni
*CI*: Confidence interval
Ct: Cycle threshold
DEC: Diarrheagenic *Escherichia coli*
DNA: Deoxyribonucleic acid
EAEC: Enteroaggregative *Escherichia* *coli*
EHEC: Enterohemorrhagic *Escherichia* *coli*
EIEC: Enteroinvasive *Escherichia coli*
EPEC: Enteropathogenic *Escherichia* *coli*
ETEC: Enterotoxigenic* Escherichia* *coli*
GBD: Global Burden of Disease
ICR: Interaction contrast ratio
IQR: Interquartile range
Lasso: Absolute shrinkage and selection operator
LightGBM: Light gradient boosting machine
MI: Multiple imputation
MNLS: Mean normalized least squares
Mul: Multiplicative interaction
NoVs: Norovirus
NoVs GII: Norovirus GII
NTS: Non-typhoidal S*almonella* spp.
*OR*: Odd ratio
PLS: Partial least squares
qPCR: Real-time fluorescence quantitative polymerase chain reaction
RERI: Relative excess risk due to interaction
RF: Random forest
RNA: Ribonucleic acid
RT: Reverse transcription
RVA: Group A rotavirus
SD: Standard deviation
S.E.: Standard error
SVM: Support vector machine
UI: Uncertainty interval
VP: Vibrio parahaemolyticus
XGBoost: EXtreme gradient boosting
