# Supplementary material for: Co-infection and interaction of enteric pathogens in acute diarrhea among children under five years: a large-scale multicenter observational study from China
Source: Infect Dis Poverty. 2025 Dec 2;14:122. doi: 10.1186/s40249-025-01392-8 (PMC12670838; doi:10.1186/s40249-025-01392-8)
Supplement: Supplementary file 1 — Additional file 1. Table S1 All 21 enteric pathogens were tested for each individual participant using a standardized panel of laboratory techniques and diagnostic methods. Table S2. Comparison of sex, age, source, and season between diarrheal and non-diarrheal children under 5 years of age. Table S3. Model comparison of risk factors for diarrhea in children under 5 years of age based on logistic regression. Figure S1. Identification of factors associated with diarrhea in children under 5 years of age using Lasso regression. Table S4. Identification of diarrhea-associated enteric pathogens using machine learning. Table S5. Composition and comparison of enteric pathogens between diarrheal and non-diarrheal children under 5 years of age. Table S6. Comparison of single and co-infections of enteric pathogens between diarrheal and non-diarrheal children under 5 years of age. [file 40249_2025_1392_MOESM1_ESM.docx]

**Additional file 1**

**RESEARCH ARTICLE**

**Co-infection and interaction of enteric pathogens in acute diarrhea among children under five years: A large-scale multi-center observational study from China**

Shun-Xian Zhang^1#^, Qin-Yan Zuo^1#^, Jin-Xin Zheng^2#^, Ji-Chun Wang^3^, Mu-Xin Chen^2^, Yu Qin^3^, Jian Yang^3^, Shan Lv^2^, Lei Duan^2^, Li-Guang Tian^2^, Qin Liu^2^, Wen-Wen Lv^4^, Rui-Tao Liu^5^, Guang-Hua Chen^5^, Wan-Fu Xu^5^, Can-Jun Zheng^3^, Shi-Zhu Li^2^* and Hong-Li Wang^5^*

^1^ Longhua Hospital, Shanghai University of Traditional Chinese Medicine, Shanghai 200032, China.

^2^ National Institute of Parasitic Diseases, Chinese Center for Disease Control and Prevention(Chinese Center for Tropical Diseases Research); National Key Laboratory of Intelligent Tracking and Forecasting for Infectious Diseases; NHC Key Laboratory of Parasite and Vector Biology; WHO Collaborating Centre for Tropical Diseases; National Center for International Research on Tropical Diseases, Ministry of Science and Technology, Shanghai 200025, China.

^3^ National Key Laboratory of Intelligent Tracking and Forecasting for Infectious Diseases, Chinese Center for Disease Control and Prevention, Beijing 102206, China.

^4^ Clinical Research Institute, Shanghai Jiao Tong University School of Medicine, Shanghai 200025, China.

^5^ Guangzhou Women and Children’s Medical Center, Guangzhou Medical University, Guangzhou 510623, People’s Republic of China.

^#^ Shun-Xian Zhang, Qin-Yan Zuo, and Jin-Xin Zheng and contributed equally to this work.

* Corresponding author: Shi-Zhu Li, [lisz@nipd.chinacdc.cn,](mailto:lisz@nipd.chinacdc.cn,)

Hong-Li Wang, [2019760935@gzhmu.edu.cn](mailto:2019760935@gzhmu.edu.cn)

**Items**

Table S1: All 21 enteric pathogens were tested for each individual participant using a standardized panel of laboratory techniques and diagnostic methods.

Table S2. Comparison of sex, age, source, and season between diarrheal and non-diarrheal children under 5 years of age.

Table S3. Model comparison of risk factors for diarrhea in children under 5 years of age based on logistic regression.

Figure S1. Identification of factors associated with diarrhea in children under 5 years of age using Lasso regression.

Table S4. Identification of diarrhea-associated enteric pathogens using machine learning.

Table S5. Composition and comparison of enteric pathogens between diarrheal and non-diarrheal children under 5 years of age.

Table S6. Comparison of single and mixed infections of enteric pathogens between diarrheal and non-diarrheal children under 5 years of age.

**Table S1** All 21 enteric pathogens were tested for each individual participant using a standardized panel of laboratory techniques and diagnostic methods.

| Pathogen | Bacterial  culture | System  biochemistry | Serological  identification | Real-time  PCR | Reverse Transcription  PCR | Reference |
| --- | --- | --- | --- | --- | --- | --- |
| **Bacteria** |  |  |  |  |  |  |
| DEC |  |  |  |  |  | [1] |
| EPEC | √ |  |  | √ |  | [1] |
| EAEC | √ |  |  | √ |  | [1] |
| ETEC | √ |  |  | √ |  | [1] |
| EHEC | √ |  |  | √ |  | [1] |
| EIEC | √ |  |  | √ |  | [1] |
| Non-typhoidal S*almonella* | √ | √ | √ | √ |  | [1] |
| *Shigella* | √ | √ | √ | √ |  | [1] |
| *Plesiomonas* | √ | √ | √ | √ |  | [1] |
| *Vibrio parahaemolyticus* | √ | √ | √ | √ |  | [1] |
| *Vibrio cholerae* | √ | √ | √ | √ |  | [1] |
| *Aeromonas* | √ | √ | √ | √ |  | [1] |
| *Clostridium difficile* |  |  |  | √ |  | [2] |
| *Campylobacter jejuni* |  |  |  | √ |  | [3] |
| *Listeria monocytogenes* |  |  |  | √ |  | [4] |
| *Yersinia enterocolitica* |  |  |  | √ |  | [4] |
| **Virus** |  |  |  |  |  |  |
| Group A rotavirus |  |  |  | √ | √ | [5] |
| Norovirus GII |  |  |  | √ | √ | [5] |
| Norovirus GI |  |  |  | √ | √ | [5] |
| Sapovirus |  |  |  | √ | √ | [6] |
| Adenovirus |  |  |  | √ | √ | [7] |
| Astrovirus |  |  |  | √ |  | [7] |
| **Intestinal protozoa** |  |  |  |  |  |  |
| *Blastocystis hominis* |  |  |  | √ |  | [8] |
| *Entamoeba histolytica* |  |  |  | √ |  | [8] |
| *Giardia lamblia* |  |  |  | √ |  | [9] |
| *Cryptosporidium* |  |  |  | √ |  | [9] |

Notes: For *Shiga* toxin-producing EIEC, we used the bioMérieux biochemical automated culture medium for differentiation. ‘√’ indicates that this diagnostic method was applied for the detection of the corresponding pathogen. Abbreviations: DEC: diarrheagenic *Escherichia coli.* EAEC: Enteroaggregative Escherichia coli. EPEC: Enteropathogenic *Escherichia coli.* EHEC: Enterohemorrhage *Escherichia coli.* EIEC: Enteroinvasive *Escherichia coli.* ETEC: Enterotoxigenic *Escherichia coli. polymerase chain reaction. PCR:* polymerase chain reaction.

Reference

[1]. Zhang, S.X., et al., Impact of co-infections with enteric pathogens on children suffering from acute diarrhea in southwest China. Infect Dis Poverty, 2016. 5(1): p. 64.

[2]. Luna, R.A., et al., Rapid stool-based diagnosis of Clostridium difficile infection by real-time PCR in a children's hospital. J Clin Microbiol, 2011. 49(3): p. 851-7.

[3]. Li, L.L., et al., Aetiology of diarrhoeal disease and evaluation of viral-bacterial coinfection in children under 5 years old in China: a matched case-control study. Clin Microbiol Infect, 2016. 22(4): p. 381.e9-381.e16.

[4]. Liu, Y., et al., Detection of 12 Common Food-Borne Bacterial Pathogens by TaqMan Real-Time PCR Using a Single Set of Reaction Conditions. Front Microbiol, 2019. 10: p. 222.

[5]. Cannon, J.L., et al., Genetic and Epidemiologic Trends of Norovirus Outbreaks in the United States from 2013 to 2016 Demonstrated Emergence of Novel GII.4 Recombinant Viruses. J Clin Microbiol, 2017. 55(7): p. 2208-2221.

[6]. Oka, T., et al., Broadly reactive real-time reverse transcription-polymerase chain reaction assay for the detection of human sapovirus genotypes. J Med Virol, 2019. 91(3): p. 370-377.

[7]. Wolffs, P.F., et al., Replacing traditional diagnostics of fecal viral pathogens by a comprehensive panel of real-time PCRs. J Clin Microbiol, 2011. 49(5): p. 1926-31.

[8]. El, S.D., et al., Prevalence, risk factors for infection and subtype distribution of the intestinal parasite Blastocystis sp. from a large-scale multi-center study in France. BMC Infect Dis, 2016. 16(1): p. 451.

[9]. Verweij, J.J., et al., Simultaneous detection of Entamoeba histolytica, Giardia lamblia, and Cryptosporidium parvum in fecal samples by using multiplex real-time PCR. J Clin Microbiol, 2004. 42(3): p. 1220-3.

**Table S2** Comparison of sex, age, source, and season between diarrheal and non-diarrheal children under 5 years of age.

| Variables |  | Non-diarrhea subjects *n* = 400  *n* (%) | Acute diarreha cases *n* = 670  *n* (%) | χ^2^ | *P* |
| --- | --- | --- | --- | --- | --- |
| Age(month, medium(Q1, Q3)) |  | 32(10,51) | 14(8, 26) | 8.201 | <0.001 |
| Subject source | Outpatients | 325(81.3) | 534(79.7) | 0.379 | 0.538 |
| Gender | Female | 141(35.3) | 228(34) | 0.165 | 0.685 |
| Season |  |  |  | 2.311 | 0.511 |
|  | Spring | 91(22.8) | 166(24.8) |  |  |
|  | Summer | 147(36.8) | 216(32.2) |  |  |
|  | Autumn | 92(23.0) | 162(24.2) |  |  |
|  | Winter | 70(17.5) | 126(18.8) |  |  |

**Table S3** Model comparison of risk factors for diarrhea in children under 5 years of age based on logistic regression.

| Variables | Area (95% *CI*) | S.E. | *P* |
| --- | --- | --- | --- |
| Age | 0.650(0.613, 0.686) | 0.019 | <0.001 |
| Diarrheagenic *Escherichia coli* | 0.518(0.482, 0.553) | 0.018 | 0.336 |
| *Vibrio parahaemolyticus* | 0.511(0.476, 0.547) | 0.018 | 0.541 |
| *Clostridium difficile* | 0.523(0.487, 0.558) | 0.018 | 0.216 |
| Group A rotavirus | 0.597(0.563, 0.631) | 0.017 | <0.001 |
| Norovirus GII | 0.534(0.499, 0.571) | 0.018 | 0.059 |
| All model | 0.733(0.701, 0.764) | 0.016 | <0.001 |

Notes: In the study, variable selection was performed using stepwise regression. Subsequently, logistic regression was conducted for each independent variable remaining in the equation to obtain the area under the curve. Finally, all variables were included in the equation to obtain the area under the curve for the final model.

Abbreviations: *CI*s: Confdence intervals. OR: Odds ratio. S.E.: Standard error.


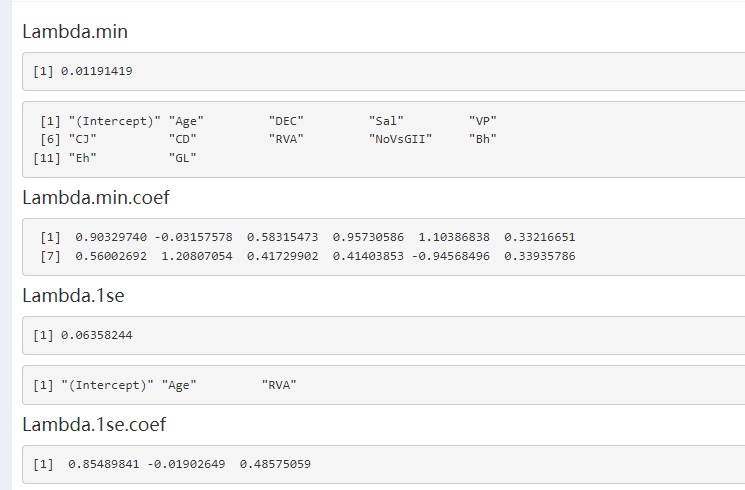


A


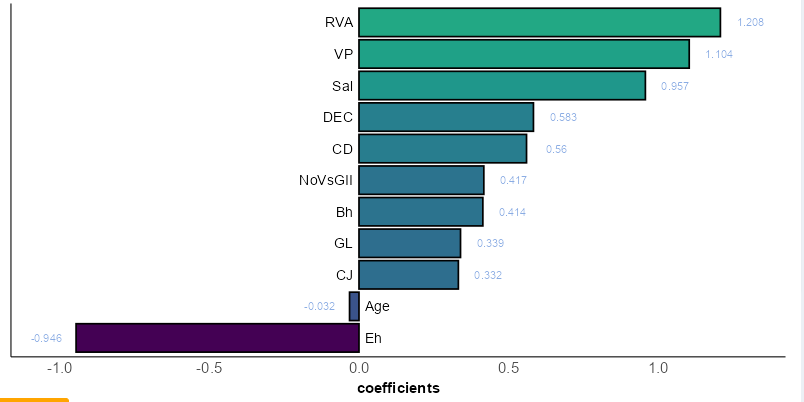


B


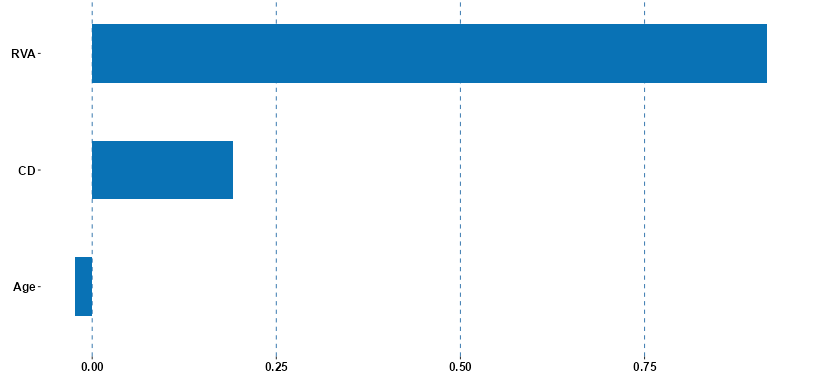


C

**Fig S1.** Identification of factors associated with diarrhea in children under 5 years of age using Lasso regression (A: Tuning parameters used for variable selection in Lassoregression. B: Variables selected by Lasso regression. C: Key variables identified by Lasso regression).

Abbreviations: Lasso: absolute shrinkage and selection operator.

**Table S4** Identification of diarrhea-associated enteric pathogens using machine learning.

| Model | Dataset | diarrhea | Non-diarrhea | Importance | AUROC (95% *CI*) | Accuracy (95% *CI*) | Sensitivity | Specificity | Recall | F1 |
| --- | --- | --- | --- | --- | --- | --- | --- | --- | --- | --- |
| CART | training set | 557 | 222 | Age=48.209 RVA =22.721 | 0.718(0.662, 0.776) | 0.718(0.684, 0.750) | 0.734 | 0.682 | 0.734 | 0.78R6 |
| CART | training set | 558 | 223 |  | 0.718(0.662, 0.777) | 0.718(0.684, 0.751) | 1.734 | 1.682 | 1.734 | 0.78R7 |
| Random forest | training set | 458 | 291 | Age=32.881 RVA=19.775 CD=14.313 SH=10.963 Aer=6.377 NoVs GII=5.948 | 0.778(0.744, 0.812) | 0.745(0.712, 0.776) | 0.877 | 0.536 | 0.877 | 0.808 |
| Random forest | test set | 212 | 109 |  | 0.723(0.662, 0.785) | 0.729(0.678, 0.777) | 0.82 | 0.558 | 0.801 | 0.801 |
| xgboost | training set | 458 | 291 | Age=0.452 CD=0.075 RVA=0.073 DEC=0.072 NoVs GII=0.071 Bh=0.068 | 0.795(0.762, 0.827) | 0.749(0.716, 0.781) | 0.812 | 0.651 | 0.812 | 0.789 |
| xgboost | test set | 212 | 109 |  | 0.754(0.697, 0.827) | 0.901(0.661, 0.754) | 0.745 | 0.615 | 0.745 | 0.767 |
| SVM | training set | 458 | 291 | Age=0.781 RVA=0.502 CD=0.287 SH=0.203 NoVsGII=0.145 Bh=0.135 | 0.739(0.702, 0.776) | 0.759(0.782, 0.792) | 0.702 | 0.585 | 0.702 | 0.744 |
| SVM | test set | 212 | 109 |  | 0.713(0.652, 0.775) | 0.762(0.714, 0.713) | 0..746 | 0.586 | 0.748 | 0.707 |
| LightGBM | training set | 458 | 291 | Age=0.916 RVA =0.083 | 0.738(0.702, 0.774) | 0.701(0.665, 0.733) | 0.782 | 0.571 | 0.782 | 0.771 |
| LightGBM | test set | 212 | 109 |  | 0.724(0.663, 0.785) | 0.689(0.635, 0.738) | 0.736 | 0.594 | 0.736 | 0.767 |

Notes: Xgboost: booster = "gbtree", eta = "0.3", gamma = "0.001", max_depth = "2", subsample = "0.7", colsample_bytree = "0.4",

Abbreviations: Aer: *Aeromonas*. AUROC: area under the receiver operating characteristic curve. Bh: *Blastocystis hominis.* CART: Classification and Regression Tree . CD: *Clostridium difficile. CI*s: Confidence intervals. DEC: diarrheagenic *Escherichia coli. LightGBM:* light gradient boosting machine*.* NoVs: Norovirus. RVA: group A rotavirus. SH: *Shigella.* SVM: support vector machine. XGBoost: eXtreme gradient boosting.

**Table S5** Composition and comparison of enteric pathogens between diarrheal and non-diarrheal children under 5 years of age

| Co-infection | | Non-diarrhea children *n* = 400 *n* (%) | Acute diarrhea children *n* = 670 *n* (%) | χ^2^ | *P* | *OR* (95%*CI*) |
| --- | --- | --- | --- | --- | --- | --- |
| Co-infection of  all pathogens | Negative | 382(95.5) | 562(83.9) | 32.592 | <0.001 | 1 (Reference) |
|  | Positive | 18(4.5) | 108(16.1) |  |  | 4.078(2.435, 6.830) |
| Co-infection of pathogen  kinds ^a^ | None | 289(72.3) | 311(46.4) | 74.852 | <0.001 | 1 (Reference) |
|  | One kind | 93(23.3) | 251(37.5) |  |  | 2.508(1.883, 3.341) |
|  | Two kinds | 16(4.0) | 87(13) |  |  | 5.053(2.896, 8.817) |
|  | Three kinds | 2(0.5) | 16(2.4) |  |  | 7.434(1.695, 32.614) |
|  | Four kinds | 0(0.0) | 4(0.6) |  |  | － |
|  | Five kinds | 0(0.0) | 1(0.1) |  |  | － |
| Co-infection of two  pathogen | No | 384(96.0) | 583(87.0) | 23.234 | <0.001 | 3.581(2.069,6.198) |
|  | Yes | 16(4.0) | 87(13.0) |  |  |  |
| Co-infection of three  pathogen | No | 398(99.5) | 654(97.6) | 5.398 | 0.020 | 4.869(1.114,21.286) |
|  | Yes | 2(0.5) | 16(2.4) |  |  |  |
| Co-infection of four  pathogen ^a^ | No | 400(100.0) | 666(99.4) | － | 0.303 |  |
|  | Yes | 0(0.0) | 4(0.6) |  |  |  |
| Co-infection of five  pathogen ^a^ | No | 400(100.0) | 669(99.9) | － | 0.999 |  |
|  | Yes | 0(0.0) | 1(0.1) |  |  |  |
| Bacterial pathogen ^a^ | None | 347(86.8) | 514(76.7) | 19.222 | 0.001 | 1 (Reference) |
|  | One kind | 51(12.8) | 140(20.9) |  |  | 1.853(1.308, 2.625) |
|  | Two kinds | 1(0.3) | 14(2.1) |  |  | 9.451(1.237, 72.203) |
|  | Three kinds | 1(0.3) | 1(0.1) |  |  | 0.675(0.042, 10.829) |
|  | Four kinds | 0(0.0) | 1(0.1) |  |  |  |
| Viral pathogen ^a^ | None | 350(87.5) | 441(65.8) | 62.76 | <0.001 | 1 (Reference) |
|  | One kind | 46(11.5) | 190(28.4) |  |  | 3.278(2.307, 4.658) |
|  | Two kinds | 4(1.0) | 37(5.5) |  |  | 7.341(2.592, 20.793) |
|  | Three kinds | 0(0.0) | 2(0.3) |  |  | － |
| Parasitic pathogen | None | 381(95.3) | 624(93.1) | 0.015 | 0.904 | 1 (Reference) |
|  | One kind | 17(4.3) | 43(6.4) |  |  | 1.544(0.868, 2.747) |
|  | Two kinds | 2(0.5) | 3(0.4) |  |  | 0.916(0.152, 5.506) |
| Co-infection of bacteria and bacteria | No | 398(99.5) | 654(97.6) | 5.399 | 0.021 | 1 (Reference) |
|  | Yes | 2(0.5) | 16(2.4) |  |  | 4.869(1.114, 21.286) |
| Co-infection of viruses  and viruses | No | 396(99) | 631(94.2) | 15.092 | <0.001 | 1 (Reference) |
|  | Yes | 4(1.0) | 39(5.8) |  |  | 6.119(2.170, 17.254) |
| Co-infection of parasites  and parasites | No | 398(99.5) | 667(99.6) | 0.015 | 0.904 | 1 (Reference) |
|  | Yes | 2(0.5) | 3(0.4) |  |  | 0.895(0.149, 5.380) |
| Co-infection of bacteria  and viruses | Negative | 399(99.8) | 668(99.7) | 0.021 | 0.885 | 1 (Reference) |
|  | Positive | 1(0.3) | 2(0.3) |  |  | 1.195(0.108, 13.217) |
| Co-infection of viruses  and parasites | Negative | 398(99.5) | 664(99.1) | 0.528 | 0.471 | 1 (Reference) |
|  | Positive | 2(0.5) | 6(0.9) |  |  | 0.895(0.149, 5.380) |
| Co-infection of bacteria  and parasites | Negative | 396(99) | 652(97.3) | 3.538 | 0.061 | 1 (Reference) |
|  | Positive | 4(1.0) | 18(2.7) |  |  | 0.895(0.149, 5.380) |
| Co-infection of bacteria, viruses, and parasites | Negative | 400(100) | 670(100) | － | － | 1 (Reference) |
|  | Positive | 0(0.0) | 0(0.0) |  |  | － |

Notes: a: Likelihood-ratio chi-square. ‘－’: it indicates that data could not be calculated.

Abbreviations: *CI*s: Confdence intervals. *ORs*: Odds ratios.

**Table S6** Comparison of single and mixed infections of enteric pathogens between diarrheal and non-diarrheal children under 5 years of age.

| Variables | Non-diarrhea subjects *n* = 400 *n* (%) | Acute diarreha cases *n* = 670 *n* (%) | χ^2^ | *P* | *OR (95% CI)* |
| --- | --- | --- | --- | --- | --- |
| single diarrheagenic *Escherichia coli* | 14(3.5) | 22(3.3) | 0.036 | 0.849 | 0.936(0.473, 1.851) |
| single Non-typhoidal Salmonella | 1(0.3) | 6(0.9) | 1.606 | 0.205 | 3.605(0.432, 30.057) |
| single S*higella* | 1(0.3) | 4(0.6) | 0.648 | 0.421 | 2.396(0.267, 21.516) |
| single *Vibrio cholerae* | 0(0.0) | 0(0.0) | － | － | － |
| single *Vibrio parahaemolyticus* | 3(0.8) | 14(2.1) | 2.874 | 0.090 | 2.824(0.807, 9.889) |
| single *Plesiomonas* | 2(0.5) | 3(0.4) | 0.015 | 0.904 | 0.895(0.149, 5.380) |
| single *Aeromonas* | 7(1.8) | 4(0.6) | 3.272 | 0.071 | 0.337(0.098, 1.159) |
| single *Yersinia enterocolitica* | 4(1) | 5(0.7) | 0.193 | 0.661 | 0.744(0.199, 2.788) |
| Single *Listeria monocytogenes* | 0(0.0) | 0(0.0) | - | - | - |
| single *Campylobacter jejuni* | 4(1) | 9(1.3) | 0.246 | 0.621 | 1.348(0.412, 4.406) |
| single *Clostridium difficile* | 6(1.5) | 18(2.7) | 1.608 | 0.205 | 1.813(0.714, 4.606) |
| single group A rotavirus | 22(5.5) | 110(16.4) | 27.607 | <0.001 | 3.375(2.097, 5.432) |
| single Norovirus GII | 10(2.5) | 27(4) | 1.756 | 0.185 | 1.638(0.784, 3.420) |
| single Norovirus GII | 1(0.3) | 0(0.0) | 1.677 | 0.195 | － |
| single Sapovirus | 0(0.0) | 0(0.0) | － | － | － |
| single Adenovirus | 6(1.5) | 8(1.2) | 0.182 | 0.671 | 0.794(0.273, 2.304) |
| single Astrovirus | 0(0.0) | 0(0.0) | － | － | － |
| single *Blastocystis hominis* | 11(2.8) | 18(2.7) | 0.004 | 0.951 | 0.976(0.456, 2.089) |
| single *Entamoeba histolytica* | 0(0.0) | 0(0.0) | － | － | － |
| single *Giardia lamblia* | 0(0.0) | 0(0.0) | － | － | － |
| single *Cryptosporidium* | 1(0.3) | 3(0.4) | 0.263 | 0.608 | 1.795(0.186, 17.311) |
| DEC_VP_PLE_LIS_Bh | 0(0.0) | 1(0.1) | 0.598 | 0.44 | － |
| DEC_CD_AD_Eh | 0(0.0) | 1(0.1) | 0.598 | 0.44 | － |
| DEC_RVA_NoVs GII_Ad | 0(0.0) | 1(0.1) | 0.598 | 0.44 | － |
| SH_LIS_RVA_Bh | 0(0.0) | 1(0.1) | 0.598 | 0.44 | － |
| PLE_CD_RVA_NoVs GII | 0(0.0) | 1(0.1) | 0.598 | 0.44 | － |
| DEC_RVA_NoVs GII | 0(0.0) | 4(0.6) | 2.397 | 0.122 | － |
| DEC_CD_GL | 0(0.0) | 1(0.1) | 0.598 | 0.44 | － |
| DEC_CD_Bh | 0(0.0) | 1(0.1) | 0.598 | 0.44 | － |
| DEC_VP_Bh | 0(0.0) | 1(0.1) | 0.598 | 0.44 | － |
| DEC_SAL_Bh | 0(0.0) | 1(0.1) | 0.598 | 0.44 | － |
| DEC_CD_NoVs GII | 0(0.0) | 1(0.1) | 0.598 | 0.44 | － |
| DEC_CJ_RVA | 0(0.0) | 1(0.1) | 0.598 | 0.44 | － |
| DEC_VP_PLE | 0(0.0) | 1(0.1) | 0.598 | 0.44 | － |
| AER_Ad_Bh | 1(0.3) | 0(0.0) | 1.677 | 0.195 | － |
| RVA_NoVs GII_Ad | 0(0.0) | 1(0.1) | 0.598 | 0.44 | － |
| CD_RVA_NoVs GII | 0(0.0) | 2(0.3) | 1.196 | 0.274 | － |
| SAL_RVA_NoVs GII | 0(0.0) | 1(0.1) | 0.598 | 0.44 | － |
| SAL_YST_CD | 1(0.3) | 0(0) | 1.677 | 0.195 | － |
| SAL_CJ_NoVs GII | 0(0.0) | 1(0.1) | 0.596 | 0.44 | － |
| DEC_NTS | 0(0.0) | 1(0.1) | 0.598 | 0.44 | － |
| DEC_VP | 0(0.0) | 3(0.4) | 1.796 | 0.18 | － |
| DEC_PLE | 0(0.0) | 3(0.4) | 1.796 | 0.18 | － |
| DEC_LIS | 0(0.0) | 1(0.1) | 0.598 | 0.44 | － |
| DEC_CJ | 0(0.0) | 2(0.3) | 1.196 | 0.274 | － |
| DEC_CD | 1(0.3) | 5(0.7) | 1.106 | 0.293 | 3.000(0.349, 25.770) |
| DEC_RVA | 1(0.3) | 12(1.8) | 4.956 | 0.026 | 7.277(0.943, 56.174) |
| DEC_NoVs GII | 1(0.3) | 8(1.2) | 2.676 | 0.102 | 4.822(0.601, 38.695) |
| DEC_Ad | 0(0.0) | 2(0.3) | 1.196 | 0.274 | － |
| DEC_Bh | 0(0.0) | 7(1) | 4.207 | 0.04 | － |
| DEC_Eh | 0(0.0) | 1(0.1) | 0.598 | 0.44 | － |
| DEC_GL | 0(0.0) | 2(0.3) | 1.196 | 0.274 | － |
| DEC_Cry | 0(0.0) | 1(0.1) | 0.598 | 0.44 | － |
| NTS_YST | 1(0.3) | 0(0.0) | 1.677 | 0.195 | － |
| NTS_CJ | 0(0.0) | 1(0.1) | 0.598 | 0.44 | － |
| NTS_CD | 1(0.3) | 0(0.0) | 1.677 | 0.105 | － |
| NTS_RVA | 0(0.0) | 2(0.3) | 1.196 | 0.274 | － |
| NTS_NoVs GII | 0(0.0) | 2(0.3) | 1.196 | 0.274 | － |
| NTS_Bh | 0(0.0) | 1(0.1) | 0.598 | 0.44 | － |
| NTS_Eh | 1(0.3) | 0(0.0) | 1.677 | 0.195 | － |
| SH_LIS | 0(0.0) | 1(0.1) | 0.598 | 0.44 | － |
| SH_CJ | 0(0.0) | 1(0.1) | 0.598 | 0.44 | － |
| SH_RVA | 0(0.0) | 2(0.3) | 1.196 | 0.274 | － |
| SH_NoVs GII | 0(0.0) | 1(0.1) | 0.598 | 0.44 | － |
| SH_Bh | 0(0.0) | 1(0.1) | 0.598 | 0.44 | － |
| VP_PLE | 0(0.0) | 2(0.3) | 1.196 | 0.274 | － |
| VP_LIS | 0(0.0) | 1(0.1) | 0.598 | 0.44 | － |
| VP_RVA | 0(0.0) | 1(0.1) | 0.598 | 0.44 | － |
| VP_NoVs GII | 0(0.0) | 1(0.1) | 0.598 | 0.44 | － |
| VP_Ad | 0(0.0) | 1(0.1) | 0.598 | 0.44 | － |
| VP_Bh | 0(0.0) | 2(0.3) | 1.196 | 0.274 | － |
| Ple_LIS | 0(0.0) | 1(0.1) | 0.598 | 0.44 | － |
| Ple_CD | 0(0.0) | 1(0.1) | 0.598 | 0.44 | － |
| Ple_RVA | 0(0.0) | 1(0.1) | 0.598 | 0.44 | － |
| Ple_NoVs GII | 0(0.0) | 1(0.1) | 0.598 | 0.44 | － |
| Ple_Bh | 0(0.0) | 1(0.1) | 0.598 | 0.44 | － |
| Aer_RVA | 0(0.0) | 2(0.3) | 1.196 | 0.274 | － |
| Aer_Ad | 1(0.3) | 0(0) | 1.677 | 0.195 | － |
| Aer_Bh | 1(0.3) | 1(0.1) | 0.136 | 0.712 | 0.596 (0.037, 9.562) |
| Aer_GL | 0(0.0) | 1(0.1) | 0.598 | 0.44 | － |
| Yst_CD | 1(0.3) | 0(0.0) | 1.677 | 0.195 | － |
| Yst_NoVsGII | 1(0.3) | 1(0.1) | 0.136 | 0.712 | 0.596(0.037, 9.562) |
| LIS_RVA | 0(0.0) | 1(0.1) | 0.598 | 0.441 | － |
| LIS_Bh | 1(0.3) | 2(0.3) | 0.021 | 0.885 | 1.195(0.108, 13.217) |
| CJ_RVA | 1(0.3) | 2(0.3) | 0.021 | 0.885 | 1.195(0.108, 13.217) |
| CJ_NoVs GII | 0(0.0) | 2(0.3) | 1.196 | 0.274 | － |
| CD_RVA | 0(0.0) | 17(2.5) | 10.313 | <0.001 | － |
| CD_NoVs GII | 1(0.3) | 6(0.9) | 1.606 | 0.205 | 3.605(0.432, 30.057) |
| CD_Ad | 0(0.0) | 1(0.1) | 0.598 | 0.44 | － |
| CD_Bh | 1(0.3) | 3(0.4) | 0.263 | 0.608 | 1.795(0.186, 17.311) |
| CD_Eh | 0(0.0) | 1(0.1) | 0.598 | 0.44 | － |
| CD_GL | 0(0.0) | 3(0.4) | 1.796 | 0.181 | － |
| RVA_NoVs GII | 2(0.5) | 33(4.9) | 15.503 | <0.001 | 10.309(2.460, 43.199) |
| RVA_Ad | 2(0.5) | 8(1.2) | 1.303 | 0.254 | 2.405(0.508, 11.381) |
| RVA_Bh | 0(0.0) | 1(0.1) | 0.598 | 0.44 | － |
| NoVs GII_Ad | 0(0.0) | 2(0.3) | 1.196 | 0.274 | － |
| NoVs GII_Bh | 1(0.3) | 3(0.4) | 0.263 | 0.608 | 1.795(0.186, 17.311) |
| Ad_Bh | 1(0.3) | 1(0.1) | 0.136 | 0.712 | 0.596(0.037, 9.562) |
| Ad_Eb | 0(0.0) | 1(0.1) | 0.598 | 0.44 | － |
| Bh_GL | 2(0.5) | 3(0.4) | 0.015 | 0.904 | 0.895(0.149, 5.380) |

Notes: ‘－’: it indicates that data could not be calculated.

Abbreviations: Ad: Adenovirus. AER: *Aeromonas.* As:Astrovirus. Bh: *Blastocystis hominis.* CD: *Clostridium difficile. CI*s: Confdence intervals. CJ: *Campylobacter jejuni.* DEC: diarrheagenic *Escherichia coli*

Eh: *Entamoeba histolytica.* GL: *Giardia lamblia.* LIS: *Listeria monocytogenes.* NoVs: Norovirus

NTS: Non-typhoidal *Salmonella. ORs*: Odds ratios. PLE: Plesiomonas. RVA: group A rotavirus. SAP: Sapovirus. SH: *Shigella.* VP: *Vibrio parahaemolyticus.* YST: *Yersinia enterocolitica*
